# Risk prediction models versus simplified selection criteria to determine eligibility for lung cancer screening: an analysis of German federal-wide survey and incidence data

**DOI:** 10.1007/s10654-020-00657-w

**Published:** 2020-06-27

**Authors:** Anika Hüsing, Rudolf Kaaks

**Affiliations:** 1 Department of Cancer Epidemiology, German Cancer Research Center (DKFZ), Heidelberg, Germany; 2 German Center for Lung Research (DZL), Translational Lung Research Center (TLRC), Heidelberg, Germany

**Keywords:** Lung cancer, Screening, Eligibility, Risk models

## Abstract

As randomized trials in the USA and Europe have convincingly demonstrated efficacy of lung cancer screening by computed tomography (CT), European countries are discussing the introduction of screening programs. To maintain acceptable cost-benefit and clinical benefit-to-harm ratios, screening should be offered to individuals at sufficiently elevated risk of having lung cancer. Using federal-wide survey and lung cancer incidence data (2008–2013), we examined the performance of four well-established risk models from the USA (PLCO_M2012_, LCRAT, Bach) and the UK (LLP_2008_) in the German population, comparing with standard eligibility criteria based on age limits, minimal pack years of smoking (or combination of total duration with average intensity) and maximum years since smoking cessation. The eligibility criterion recommended by the United States Preventive Services Taskforce (USPSTF) would select about 3.2 million individuals, a group equal in size to the upper fifth of ever smokers age 50–79 at highest risk, and to 11% of all adults aged 50–79. According to PLCO_M2012_, the model showing best concordance between numbers of lung cancer cases predicted and reported in registries, persons with 5-year risk ≥ 1.7% included about half of all lung cancer incidence in the full German population. Compared to eligibility criteria (e.g. USPSTF), risk models elected individuals in higher age groups, including ex-smokers with longer average quitting times. Further studies should address how in Germany these shifts may affect expected benefits of CT screening in terms of life-years gained versus the potential harm of age-specific increasing risk of over-diagnosis.

**Electronic supplementary material:**

The online version of this article (10.1007/s10654-020-00657-w) contains supplementary material, which is available to authorized users.

## Introduction

Following results from the earlier US National Lung Cancer Screening trial (NLST) [1], recent findings from the Dutch-Belgian NELSON trial [2] and five smaller randomized trials in Italy, Denmark and Germany [3–7] confirm that low-dose computed tomography (LDCT) screening is a viable means to reduce lung cancer mortality, eliciting plans for introducing lung cancer screening programs in European countries. For cost-effectiveness, and to ensure that expected benefits of screening clearly outweigh potential harms to radiation, false-positive diagnostic workup or over-diagnosis it is essential that screening be targeted to individuals at sufficiently elevated risk. Randomized screening trials have used simplified inclusion criteria for selecting high risk individuals, based on age limits, minimal pack years of smoking (or combination of total duration with average intensity) and maximum years since smoking cessation. In the USA and Canada, the criteria used for the NLST trial—i.e., being 55–74 years of age, having smoked over 30 pack-years, no more than 15 years since smoking cessation—formed the basis for official screening recommendations (with extension of upper age limit to 79 years) [8, 9].

An alternative to concise inclusion criteria is the use of more refined models for the prediction of an individual’s absolute lung cancer risk. Several models have been developed on the basis of an individual’s age and detailed smoking history, presence of pulmonary disease (e.g., chronic-obstructive-pulmonary-disease (COPD), emphysema), family or personal history of cancer, body-mass-index and socio-economic background indicators. Major models (reviewed in [10–12]) have been developed in context of large-scale prospective cohorts and trials, particularly in the USA and the United Kingdom (Prostate, Lung, Colorectal, and Ovarian Cancer Screening Trial [PLCO], [CARET], American Association of Retired Persons Study [NIH-AARP], NLST, Liverpool Lung Project [LLP]), and have been externally validated in independent cohorts [12–15]. Follow-up analyses in the NLST and PLCO trials [12, 16] and other population cohorts [14, 15] have shown that, at equal numbers of individuals selected as eligible as obtained with concise inclusion criteria, risk models identified more individuals actually developing lung cancer. Risk models also offer greater conceptual clarity of offering equal access to screening for individuals at equal risk [17], combined with greater flexibility towards improved risk discrimination by integrating age, smoking habits and other risk factors with genetic risk scores or other biomarkers [18, 19]. However, questions remain with regard to the accuracy of model calibration, model risk thresholds to be used as cut-off points for screening eligibility, and general health risk characteristics of subjects determined eligible by models compared to criteria.

In view of the possible introduction of lung cancer screening in Germany, we analyzed data from federal-wide health surveys and cancer registries to examine screening eligibility according to selected (NLST, USPSTF, NELSON) criteria or risk models, and to describe the risk factor profiles of individuals eligible by these different criteria as compared to those eligible by model thresholds.

## Study data, methods for statistical analyses

### German population data on smoking prevalence

The *German Health Update* study (GEDA; “Gesundheit in Deutschland aktuell”) is a health monitoring program consisting of cross-sectional surveys conducted by the Robert Koch Institute to provide data on health and disease, health determinants and health behaviors from nationally representative samples of adults in Germany [20]. Between 2008 and 2013, three GEDA studies were carried out, in 2008–2009, 2009–2010 and 2012–2103, involving a total of 62,606 computer-assisted telephone interviews. Data are available for research purposes in public use files, including individual weighting factors to allow projections towards full German adult population counts [20]. Participants completed questionnaire interviews on education, life-style and body composition, social and psychological health conditions, personal health, and use of medication and health care programs. Information on smoking includes smoking status as never, current regular, current sporadic, or former smoker, age at smoking initiation (in 5-year categories), age at quitting smoking (in 5-year categories), and smoking intensity in terms of cigarettes smoked per day (in 5-cigarette-categories). Duration of smoking, and in case of former smokers, time since quitting was derived from age at start smoking, age at recruitment (in 5-year categories) and age at stopping smoking.

### Statistical analyses

#### Coding of variables; imputation of missing data; weighting of GEDA data

In our analyses all categorical data on smoking exposure were scored quantitatively by the category mid-points. To reduce bias from analyzing incomplete data, sporadic missing values (max of 7% per variable ) were substituted with study-specific single imputation, taking the median value or most frequent category by age-group, gender, and smoking status (for smoking-related variables). Information on history of pneumonia, and family history of cancer was not available. Since these latter conditions have relatively low prevalence in the German population all participants were assumed to be disease-free, leading to slightly conservative estimates of lung cancer risks. Population weights divided by 3 to account for threefold representation of the general population by the three study waves [21] were used to derive results (population mean and percentages, and approximate absolute numbers) reflecting the average general German adult population 2008–2013.

#### Eligibility criteria for screening

Trials in the USA and Europe have used variable inclusion criteria based used different age limits, lifetime cumulative smoking history and maximum duration since smoking cessation, and put different weights on exposure duration and intensity [1, 3–6, 22]. We focused on model comparisons with the NLST/USPSTF and NELSON eligibility criteria, which empirically were at opposite ends on the scale of inclusiveness when applied to the German population (GEDA) (Supplemental Table 1). In NLST [1], eligibility was based on having a minimum age of 55, stopping at age 75, 30 or more pack-years of smoking and a maximum of 15 years since quitting (summary code: 55-75-30-15). USPSTF eligibility recommendations are identical but stopping screening at age 80 (summary code: 55-80-30-15). The European NELSON trial, the second largest randomized trial after NLST, included individuals of younger age (range 50–74 years), having smoked at least 10 cigarettes a day for 30 years (corresponding to 15 pack years) or 15 cigarettes a day for 25 years (equivalent to 18.75 pack years), but maximally only 10 years since quitting for those who had stopped smoking (50-75-10 ×  30/15 ×  255-10). Thus, compared to NLST, NELSON allows inclusion of younger subjects and puts lower weight on smoking intensity, with a stronger restriction on minimum smoking duration and on maximum time since quitting. Further to the NLST/USPSTF and NELSON criteria, we also examined the more restrictive criterion of 55-75-40-10 (starting age 55, stopping at age 75, ≥ 40 pack years, < 10 years since quitting), which in several recent simulation studies was found among the most cost-effective screening scenarios [23, 24].

#### Application of lung cancer risk models

Risk models evaluated include models for prediction of individuals’ absolute lung cancer risk that were developed in various US cohorts (PLCO_M2012_ [16], with subsequent adaption enabling risk estimation also for non-smokers PLCO_ALL2014_ [25]; Lung Cancer Risk Assessment Tool [LCRAT] [26]; model by Bach et al. on CARET cohort [27] and in the UK Liverpool Lung Project (LLP_2008_) [28, 29]. Supplemental Table 2a summarizes model variables, the design and population characteristics of the studies (prospective/case-control) in which models were first developed, and time frame for risk prediction (5-year, 6-year, other). The models differ with regard to the shape and strength of age-related increase in risk, inclusion of a gender effect (reduced risk among women compared to men, included in the BACH, LLP_2008_ and LCRAT models but not in PLCO), and variables and model coefficients accounting for smoking history (pack-years, duration in years, intensity as lifetime-average cigarettes/day, and time since quitting) (Supplemental Table 2b). Gradual risk attenuation among ex-smokers with increasing years of time since quitting is accounted for in the BACH, PLCO_M2012_ and LCRAT models, but not in LLP_2008_. Additional predictor variables include BMI and educational status (PLCO_M2012_, LCRAT models), family history of lung cancer (LLP_2008_, PLCO, LCRAT), personal history of cancer (LLP_2008_, PLCO_M2012_), pre-existing lung-disease such as pneumonia (LLP_2008_) and emphysema (LCRAT), and asbestos exposure (BACH, LLP_2008_). PLCO_ALL2014_ and LCRAT models also allow for an ethnicity-correction, which was irrelevant for our analyses for the German population which is mostly of Caucasian by origin. Supplemental Table 3 describes basic characteristics of discrimination performance and calibration in model development and replication studies.

Model estimates were generated with R-package LCMODELs (https://dceg.cancer.gov/tools/risk-assessment/lcmodels**)** and according to published parameters (PLCO_ALL2014_ results were verified with spreadsheet as provided by Tammemägi [25]). All estimates were linearly standardized to 5-year projection time-span, e.g. predictions for 6 years were multiplied by a factor 5/6. The predictive capacity of the different models was described using Lorenz-curves, showing the cumulative proportion of total cancer incidence that is predicted to occur within a progressively widening proportion of individuals in the population at risk, ranked from highest risk to the lowest. This display describes the degree to which predicted cumulative lung cancer risk is concentrated within variably restricted subsets of the population (model risk “predictiveness”), as a function of overall population variance of predicted risks [30].

## Results

### Basic description of the German population (GEDA 2008–2013)

In the three GEDA population samples from 2008 to 2013, and restricted to age range 50–79, 45% of respondents were men and 55% were women. In the full German adult population according to the re-weighted GEDA survey samples, percentages were more balanced with 53% women, and covering an average total of 14.1 million men and 15.7 million women, of whom 6.3 million current smokers and 9.6 million ex-smokers (Table 1). Adults 50–79 years represented close to 30 million people (15.7 million women and 14.1 million men). Among these, in the years 2009, 2010 and 2013 German cancer registries (https://www.krebsdaten.de/) on average reported a total of 42,800 incident cases of lung cancer per year in the full German population, representing 85% of cases in the full German population (average annual incidence of 50,500). Considering that screening eligibility will likely be limited to individuals within the latter age rage, further analyses presented below refer to the projected total population (re-weighted survey data) of German women and men age 50 to < 80.

**Table 1:** Lung cancer risk factor distribution in participants of GEDA-studies 2008–2013 by sex: participant count, average weighted population percentages (%) and gross average population count [Mio], or median (inter-quartile range [IQR]) in the general German population

Total	Men (2008-2013)	Women (2008-2013)
	28033 (100.00% , 33.2 Mio)	34573 (100%,35.2 Mio)
< 50	15064 (54.86%, 18.2 Mio)	17980 (49.93%, 17.6 Mio)
50–54	2670 (9.23%, 3.1 Mio)	3432 (8.47%, 3.0 Mio)
55–59	2198 (7.98%, 2.7 Mio)	2927 (7.80%, 2.7 Mio)
60–64	2285 (6.62%, 2.2 Mio)	2657 (6.42%, 2.3 Mio)
65–69	2118 (6.96%, 2.3 Mio)	2501 (7.19%, 2.5 Mio)
70–74	1930 (7.52%, 2.5 Mio)	2278 (8.67%, 3.0 Mio)
75–79	1049 (4.21%, 1.4 Mio)	1465 (6.08%, 2.1 Mio)
80+	719 (2.61%, 0.9 Mio)	1333 (5.45%, 1.9 Mio)
Aged 50–79	12250 (100.00% , 14.1)	15260 (100%, 15.7)
Body mass index (kg/m^2^)	24.3 (21–27)	23.1 (20–27)
Education (US coding)	2.6 (2.2–3.0)	2.3 (1.0–2.8)
COPD, Emphysema	1057 (9.04%, 1.3)	1760 (11.71%, 1.8)
Smoking: never	4264 (32.89%, 4.6 Mio)	8407 (58.7%, 9.2 Mio)
Current	2743 (24.28%, 3.4 Mio)	2988 (18.4%, 2.9 Mio)
Former	5238 (42.80%, 6.0 Mio)	3865 (22.9%, 3.6 Mio)
Ever smoker	7981 (100.00%, 9.5 Mio)	6853 (100.00%, 6.5 Mio)
Eligible by		
NLST (55-75-30-15)	1697 (21.76%, 2.1 Mio)	944 (15.15%, 1.0 Mio)
USPSTF (55-80-30-15)	1790 (23.14%, 2.2 Mio)	989 (16.07%, 1.0 Mio)
55-80-40-10	967 (12.88%, 1.2 Mio)	531 (8.72%, 0.6 Mio)
NELSON (50-75-10 cpd × 30 years /15 cpd × 25 years -10)	2649 (34.87%, 3.3 Mio)	2190 (33.80%, 2.2 Mio)

### Smoking prevalence by sex and age group

**Fig. 1:**
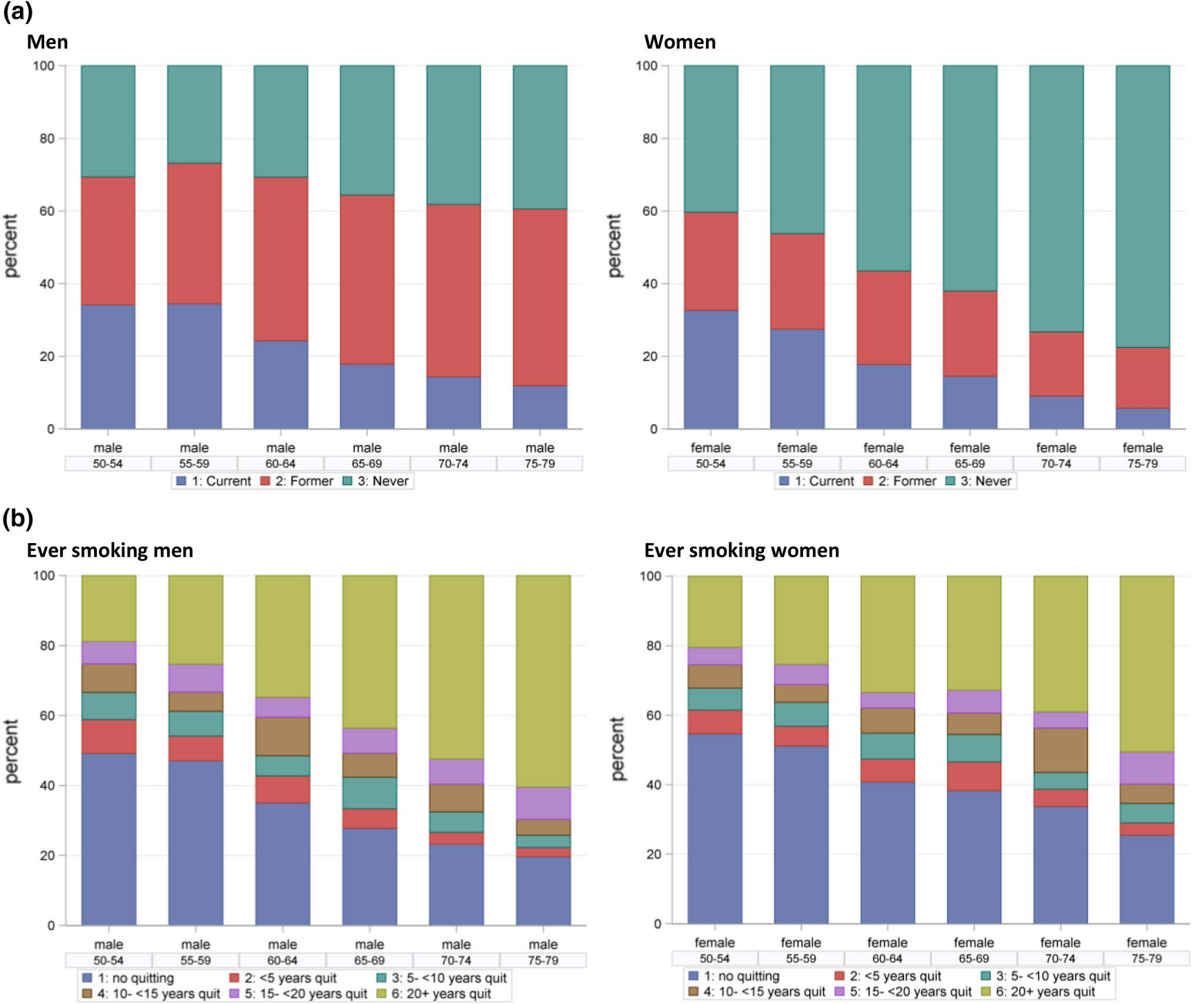
Proportion of smoking status in men and women by 5-year age-group in the German adult population (GEDA2008-2013)

Across all selected age groups 50 to < 80, 67% of men and 41% of women in the German population were ever-smokers. For both sexes, analyses by 5-year age group showed a trend towards progressively lower percentages of current smokers with increasing age (Fig. 1a): 34% of men and 33% of women reported current smoking at ages 50–54 versus 12% and 6%, respectively, at ages 75–79. As percentage of the total population, and in all age groups the percentage of never smokers was higher among women than among men. Among ever-smokers only, the percentage of ex-smokers and the duration of time since quitting increased strongly with age in both sexes (Fig. 1b). Life-time average numbers of cigarettes smoked per day and total pack-years of smoking were generally higher for men than women (Supplemental Fig. 1). There were no major differences in life-time smoking duration between ever-smoking men and women or by age group (Supplemental Fig. 1).

### Screening eligibility according to selected inclusion criteria

**Fig. 2:**
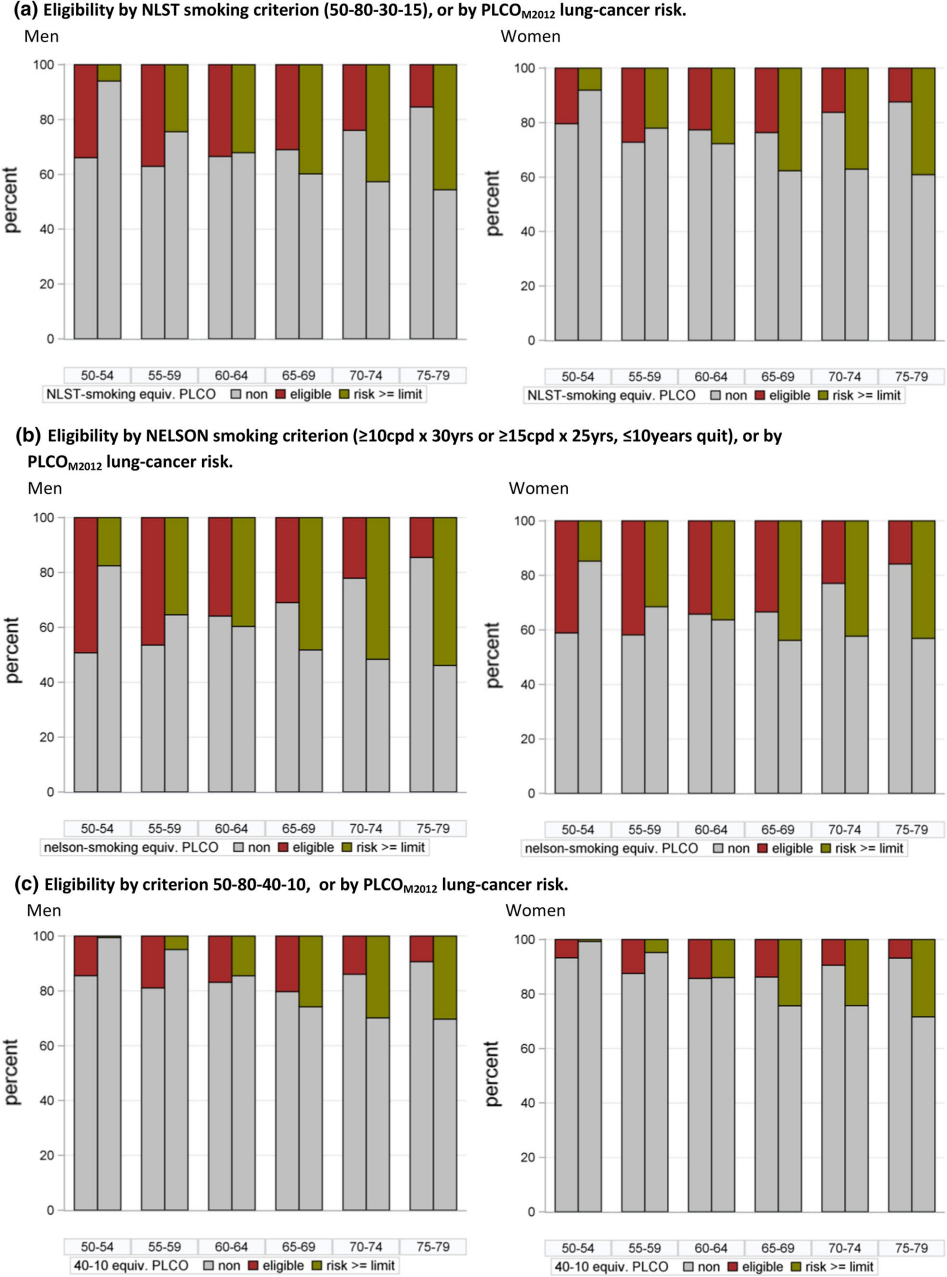
Eligibility to smoking criteria or risk estimate above threshold identifying equal number of persons among ever-smoking men and women in the German general population (according to GEDA 2008–2013)

For the combined age and smoking criteria of NLST, USPSTF and NELSON, the average percentages of screening-eligible individuals among ever-smokers aged 50–79 in GEDA were 19%, 20% and 34%, respectively, and 10% and 11% for criteria 55-75-40-10 (age 55- < 75, ≥ 40 pack-years, ≤ 10 years quit) and 55-0-40-10. Considering only the smoking component of the criteria, and examining data by sex and 5-year age group (Fig. 2), eligibility was generally higher in the younger age groups and lowest in the older age, with proportions changing from 46 to 15% for NELSON and 11–8% for the 40-10 criterion (≥ 40 pack-years, ≤ 10 years quit) from age 50–54 to 75–79, respectively. Among ever smoking men the eligible proportions for NELSON-smoking and 30- 15-, 40-10-criteria were, respectively, 49%, 34%, and 14% at 50–54 years versus 15%, 15% and 9% at age 75–79. A similar age trend was also visible in ever smoking women, with 41%, 20% and 7% identified at age 50–54 by the NELSON-smoking, 30-15- and 40-10- criteria versus 16%, 12% and 7% at age 75–79.

### Absolute risks estimated by lung-cancer risk models

Among ever-smokers, estimates for developing lung cancer within 5 years from various risk models ranged from 0.0% to a maximum of 32.2%, depending on the specific risk model, with average values of 1.4%, 2.2%, 1.25 and 1.5% for models BACH, LLP_2008,_ PLCO_M2012_ and LCRAT, respectively (Supplemental Fig. 2, box plots by sex and age group). The overall range of predicted risk (min, max) was largest for PLCO_M2012_ (0.0%–32.2%) and smallest for Bach (0.02%– 10.6%). Among never-smokers risk estimates from the PLCO_ALL2014_ model ranged from 0.0 to 1.0%, with an average of 0.2%. The distribution of 5-year risk estimates with respect to different eligibility criteria shows that individuals meeting eligibility criteria may still have relatively small risk estimates, while the majority of eligible subjects clearly have higher estimated risk than average/non-eligible persons (Supplemental Fig. 3, box plots by eligibility to different criteria).

Calculated absolute risk scores correlated perfectly (Pearson’s correlation, r = 1.00) between the PLCO_M2012_ and PLCO_ALL2014_ models, and correlated highly (r > 0.82) across all models developed in the USA (PLCO, LCRAT, BACH) but only moderately (r = 0.60–0.72) between LLP_2008_ and any of the US models (Supplemental Table 4).

### Classification potential by risk models

**Fig. 3:**
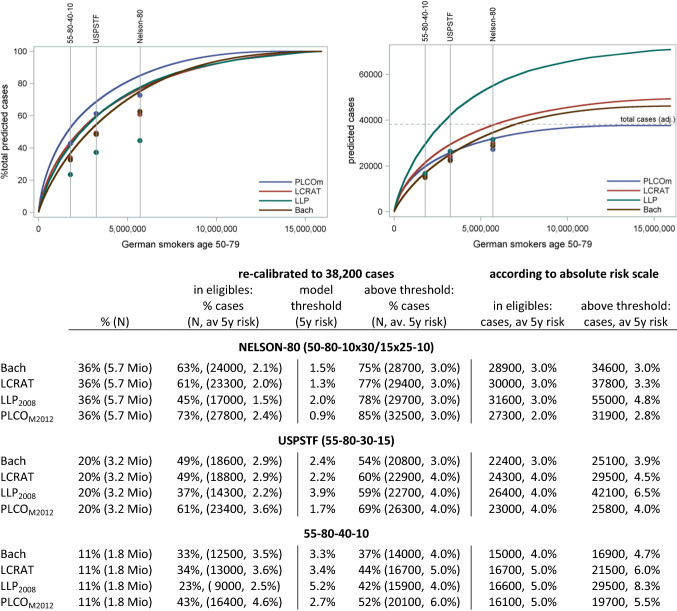
Predictive capacity of lung cancer risk models projected for the population of ever-smoking German adults age 50–79: per cent predicted incidence against population per cent classified at highest risk

Figure 3 shows the *predictiveness* of the PLCO_M2012_, LCRAT, BACH and LLP_2008_ models in German ever smokers age 50–79 (GEDA, 2008–2013) in terms of Lorenz curves, plotting predicted total lung cancer incidence for increasing population proportions of individuals at highest risk. In Fig. 3a, incidence is expressed as proportion of total incidence, whereas Fig. 3b presents curves for absolute incidence counts as predicted from models’ absolute risk estimates. The models disagree on the total number of predicted lung cancer cases (Fig. 3b), with results from the PLCO-model closest to the case number attributed to smokers. On the proportional scale, as judged by the area under the Lorenz curve (Fig. 3b) the PLCO-model-curve shows a somewhat larger population heterogeneity of predicted lung cancer risks than the other models.

According to the various Lorenz curves (Fig. 3a) models predict 54–69% of lung cancer incidence to occur in 20% of ever smokers age 50–79 at highest risk, the proportion of individuals that would be selected by the USPSTF criteria (55-80-30-15), if models were used to select the highest-risk individuals. Likewise, 75–85% of incidence is predicted to occur in the 36% at highest risk, equivalent to the percent of ever smokers eligible by age-extended NELSON-like criteria (50-80-10 × 30/15 ×   25-10).

Within a more restricted age-range of 50 to < 75, the NELSON criterion would include 38% of the population, optimally including 77–85% of projected 31,700 total cases (Supplemental Fig. 4).

Compared to the USPSTF or NELSON eligibility criteria, and at equivalent specificity, all models elected more individuals in the higher age groups, including ex-smokers with longer average quitting times. For PLCO_M2012_ this is illustrated exemplarily in Fig. 2; Table 2, and in Supplemental Fig. 5 and Supplemental Table 5.

**Table 2:** Personal features of ever-smoker age 50–79 selected by USPSTF eligibility criteria or by risk-threshold identifying same number of subjects to be screened, or both

% (N[Mio]) or mean (min–max)	USPSTF+, PLCO_M2012_− risk < 1.7%	USPSTF−, PLCO_M2012_− risk ≥ 1.7%	USPSTF+, PLCO_M2012_- risk ≥ 1.7%
	100% (0.9 Mio)	100% (1.0 Mio)	100% (2.3 Mio)
Male	68% (0.6 Mio)	59% (0.6 Mio)	68% (1.5 Mio)
Female	32% (0.3 Mio)	41% (0.4 Mio)	32% (0.7 Mio)
Current smoker	42% (0.4 Mio)	44% (0.4 Mio)	66% (1.5 Mio)
Former smoker	58% (0.6 Mio)	56% (0.5 Mio)	34% (0.8 Mio)
COPD/emphysema	6% (0.1 Mio)	25% (0.2 Mio)	20% (0.5 Mio)
Age (years)	59.2 (57.5–72.5)	70.7 (52.5–77.5)	66.4 (57.5–77.5)
Below age 55	0	10% (0.1 Mio)	0
Smoking history [ys]	37.3 (16.0–50.5)	40.7 (17.5–65.5)	46.5 (27.5–70.1)
Lifetime average	22.8 (13.0–86.5)	22.0 (5.0–100)	24.0 (10.0–120)
Cigarettes per day			
Lifetime cigarette consumption (packyears)	41.6 (30.0–156)	40.1 (8.8–147)	54.8 (30.0–240)
Years since quitting	4.7 (0.0–14.5)	10.7 (0.0–43.5)	2.4 (0.0–14.5)
Body-mass-index (kg/m^2^)	28.9 (18.0–43.0)	26.3 (18.0–43.0)	26.5 (18.0–43.0)
Education (US coding)	3.3 (1.0–6.0)	2.4 (1.0–6.0)	2.7 (1.0–6.0)

	USPSTF+, LLP_2008_− risk < 3.9%	USPSTF−, LLP_2008_− risk ≥ 3.9%	USPSTF+, LLP_2008_− risk ≥ 3.9%
	100% (1.6 Mio)	100% (1.7 Mio)	100% (1.6 Mio)
Male	61% (1.0 Mio)	58% (1.0 Mio)	75% (1.2 Mio)
Female	39% (0.6 Mio)	42% (0.7 Mio)	25% (0.4 Mio)
Current smoker	55% (0.9 Mio)	64% (1.1 Mio)	63% (1.0 Mio)
Former smoker	45% (0.7 Mio)	36% (0.6 Mio)	37% (0.6 Mio)
COPD/emphysema	16% (0.3 Mio)	14% (0.2 Mio)	16% (0.3 Mio)
Age (years)	59.9 (57.5–77.5)	70.8 (57.5–77.5)	68.8 (57.5–77.5)
Below age 55	0	0	0
Smoking history (years)	38.7 (16.0–50.5)	46.4 (21.0–70.1)	49.0 (33.0–70.1)
Lifetime average	25.0 (12.0–120)	7.9 (0.0–70.0)	22.3 (10.0–80.0)
Cigarettes per day			
Lifetime cigarette consumption (packyears)	47.8 (30.0–240)	17.1 (0.0–147)	54.1 (30.0–196)
Years since quitting	3.7 (0.0–14.5)	5.4 (0.0–42.5)	2.5 (0.0–14.5)
Body-mass-index (kg/m^2^)	27.7 (18.0–43.0)	26.5 (18.0–43.0)	26.6 (18.0–43.0)
Education (US coding)	2.9 (1.0–6.0)	2.8 (1.0–6.0)	2.9 (1.0–6.0)

### Model projections for absolute incidence

In 2009, 2010 and 2013 German cancer registries on average reported 50,500 incident cases of lung cancer in total, of which 29,100 among men and 13,700 among women in the age range of 50–79 years, which is 85% of all cases (https://www.krebsdaten.de/). Using the PLCO_ALL2014_ model, we find that about 6% of cases in men and 21% of cases among women developed among never-smokers. Subtracting the age- and sex-specific percentages of predicted lung cancer among non-smokers from total incidence, we estimate that about 27,400 and 10,900 annual cases occurred among ever-smoking men and women respectively, for a total of 38,200 cases (76% of total lung cancer cases in Germany over all ages). Applying the proportions of total predicted lung cancer incidence in Fig. 3a to this overall case number—as a calibration-in-the-large approach [31]—allows estimation of approximate annual case numbers, as well as average 5-year risk for variable proportions of ever smokers ranked at highest risk (Fig. 3). Focusing federal-wide screening on the 20% of individuals at highest risk among smokers aged 50–79, an equivalent number as selected by USPSTF criterion, would cover between 20,800 (54%; Bach) and 26,300 (69%, PLCO_M2012_) annual cases of lung cancer, with average 5-year risk of lung cancer between 3.0% (Bach) and 4.0% (PLCO_M2012_) for the eligible population part. Criterion 55-80-40-10 would select 11% individuals, including 14,000 (Bach, 37%) to 20,100 (PLCO_M2012_, 52%) of predicted cases, if selection was by highest risk.

On the original model scales (Fig. 3b), the total predicted annual incidence of lung cancer in Germany varied almost two-fold from 37,600 cases for PLCO_M2012_ to 70,900 cases for LLP_2008_, indicating major differences in model calibration. More detailed plots of predicted versus observed incidence by sex and 5-year age group (Supplemental Fig. 6) show good fit for PLCO_ALL2014_ and PLCO_M2012_ predictions to observed incidence among both men and women, reasonable fit for predictions by LCRAT among men but over-estimation among women, modest overestimation of risks in both sexes by Bach, and major overestimation for both sexes by LLP_2008_. Compared to the incidence attributed to smokers, the predicted cumulative incidence combined was 86% higher for LLP_2008_, 21% higher for Bach, 29% higher for LCRAT and 1% lower for PLCO_M2012_. For the 20% of individuals ranked at highest-risk, average 5-year risks estimated on original model scales ranged from 3.9 and 4.0% for Bach and PLCO_M2012_ to 6.5% for LLP_2008_. For the 11% at highest risk (55-80-40-10 equivalent), average 5-year risks estimated on original model scales ranged from 4.7% for Bach, 5.5% for PLCO_M2012_ to 8.3% for LLP_2008_.

## Discussion

To maintain acceptable cost-benefit and clinical benefit-to-harm ratios LDCT screening should be offered only to individuals at sufficiently elevated risk of having lung cancer, and the definition of an appropriate minimal risk criterion, with reference to accepted instruments for its assessment, is a crucial element in setting up the regulatory framework for the introduction of screening programs.

### Assessment of absolute risk predictions (calibration in the large)

We examined the performance of eligibility criteria and risk models in German federal-wide health survey and lung cancer incidence data. Predicted risks showed high correlations between all US models, whereas the LLP_2008_ model showed a more independent assessment compared to, both, US model risk estimates and eligibility criteria. Comparisons of predicted versus observed lung cancer incidence federal-wide in Germany suggests good calibration for the PLCO_M2012_/PLCO_All2014_ models and moderately so for the Bach and LCRAT models, whereas the LLP_2008_ model appears to clearly overestimate risk. Validation analyses in various prospective cohorts in the USA and Germany have generally also shown good calibration for PLCO_M2012_ (predicted-to-observed [P/O] lung cancer incidence ratios between 0.92 and 1.15), Bach (P/O ratios of 0.88–1.03) and LCRAT (P/O ratios of 0.97–1.17), and more variable results for LLP_2008_ (P/O ratios of 0.96–1.72) [12–16] (see also Supplemental Table 3). However, caution needs to be applied especially with regard to highest risk estimates, which have in the past been found to be too extreme [12, 15, 25].

### Screening-eligible individuals by concise eligibility criteria

Projected to the overall German population (GEDA, 2008–2013 average), about one third (34%, 5.5 million) of ever smokers in the age range of 50–79 years would be eligible for screening by the NELSON criteria for both age and smoking (55-75-10 × 30/15 ×  255-10) versus about one fifth (20%; 3.2 million) by the USPSTF (55-80-30-15) criteria, and 11% by the 55-80-40-10 criteria. Based on risk models, calibrated-in-the-large to federal-wide annual lung cancer incidence (Fig. 3a), we find that among ever-smokers aged 50–79 about 39–62% (NELSON), 37–61% (USPSTF), 32–51% (NLST) and 23–43% (55-80-30-15) of the lung cancer incidence are expected to occur in individuals eligible by these respective criteria, with estimated average 5-year risks of, respectively, 1.3–2.1%, 2.2–3.6%, 2.0–3.2% and 2.5–4.6%.

### Outweighing harms: radiation risks, and invasive diagnostic work-up after false-positive detection

Two types of clinical harm that may motivate using minimal-risk criteria for LDCT screening are radiation-induced cancer risks and risk of invasive diagnostic examinations following a false-positive screen test.

Models for radiation-related cancer risks [32] predict higher risks of major cancers (e.g., lung, breast) with increasing cumulative radiation dose (total number of screens lifetime, plus radiation due to possible follow-up examinations) and with younger age at start of CT screening, higher radiation-related cancers risks among smokers, and higher risks for women than for men. Based on these models, it has been shown that for men and women 50 years and older with a recent history of at least moderately high cumulative smoking exposure (≥ 10–20 pack-years), on average the expected benefits of screening (averted lung cancer deaths) will largely outweigh possible harms due to radiation-induced tumors in both men and women [23, 33–35]. In the Italian COSMOS trial, based on detailed dosimetry data for radiation exposures Rampinelli et al. estimated that ten years of annual screening would entail a lifetime risk of radiation-induced cancers < 5 per 10,000 among men and < 10 per 10,000 among women aged 50- and older with ≥ 20 pack years of smoking [33]. Assuming at least 80% sensitivity of lung cancer detection and 20% mortality risk reduction by LDCT screening, it can be calculated that to minimally offset these lifetime risks of radiation-induced cancer screening participants should have a 10-year lung cancer risk of at least 30–60 per 10,000 (0.3–0.6%). Inspection of the box plots in Supplemental Fig. 3 shows that small proportions of individuals eligible by NELSON or USPSTF criteria may not actually reach this minimum risk level, which argues in favor of using individual model risk estimates as criterion for screening eligibility.

False-positive screen tests can cause major harm especially when they lead to further, invasive medical investigations. An analysis of NLST trial data showed that, even within the limits of NLST eligibility criteria, the ratio of true-positive lung cancer diagnoses over invasive diagnostic work-up (bronchoscopic or surgical biopsies) triggered by a false-positive screen-test can still vary substantially according to individuals’ 5-year lung cancer risk, from about 1.35 in the lowest risk deciles of the PLCO_M2012_ risk score (i.e., 5-year risk < 1.0%) to about 5.0 in the highest decile (5-year risk ≥ 6.5%) [36]. These findings indicate that the balance between expected benefit of screening (life years gained through mortality reduction, for expected true test positives) versus the risk of undergoing invasive diagnostic investigations following a false-positive screen test will depend not only on an individual’s number of screening participations, but also on a person’s actual lung cancer risk. Again, this argues in favor of using minimal risk threshold for screening, assessed by a well-calibrated risk model.

### Improved identification of highest-risk individuals: use of risk prediction models

Analyses in prospective population cohorts in North America [12, 15, 16] have shown that at equivalent specificity, the PLCO_M2012_, LCRAT and Bach models, especially, could identify individuals developing lung cancer over the next 5–6 years with up to 10 to 19% greater sensitivity than with the NLST/USPSTF criteria based on minimum age and cumulative smoking history and maximum time since quitting, and similar findings were made in cohorts in Germany [14] and Australia [13]. For the German population of ever-smokers age 50–79 we find that, at equivalent numbers of screen-eligible individuals as with USPSTF criteria, risk models would cover about 54–69% of lung cancer incidence (2000 to 8400 extra cases) among the 20% of smokers classified at highest risk, using 5-year risk thresholds ranging from 1.75% for PLCO_M2014_ to 2.32% and 2.49% for LCRAT and BACH, respectively or 3.85% for LLP_2008_. For the PLCO_M2012_, LCRAT or Bach models, comparable risk thresholds were reported in ever-smokers in the same age range in the US general population, based on data of the National Health Interview Survey (NHIS) [15] (Table 3). However, risk thresholds for inclusion of equivalent numbers of individuals as with NLST, USPSTF or other criteria may vary across populations because of different age and sex-specific distributions of individuals’ lung cancer risks or differences in the population age range considered.

**Table 3:** Estimated model risk thresholds (5-year risk) identifying highest-risk individuals at equivalent numbers as with the NLST/USPSTF eligibility criteria, in study populations in Germany (GEDA, EPIC), USA and Australia

Study	PLCOM2012	LCRAT	LLP52008	BACH
GEDA^a^ 2008–2013 (55–74 years)	1.46	1.98	2.74	2.20
GEDA^a^ 2008–2013 (50–74 years)	1.52	2.03	2.74	2.27
GEDA^a^ 2008–2013 (50–79 years)	1.75	2.32	3.85	2.49
EPIC-D^b^ 1992–2009 (40–69 years)	2.11	1.61	1.53	1.55
PLCO-CXR^c^ 19 (ever smoker age 55–74 years)	1.12		1.48	1.32
AARP^d^ (ever smoker age 50–71 years)	2.00	2.00		2.00
CPS-II^e^ (ever smoker age 40–92)	2.00	2.00		2.00
45-upf (age 55–74 years)	1.44			
NHIS^g^ (1997–2001) survey (ever smoker age 50–80 years)		1.90		
NHIS^g^ (2010–2012) survey (ever smoker age 50–80 years)	1.67	2.00		2.40

Findings from our analysis in GEDA-data and as reported for other data from the literature, if necessary standardized to 5-year risk

^a^Gesundheit in Deutschland aktuell [20]

^b^European Prospective Investigation into Cancer and Nutrition, Germany (Deutschland) [14]

^c^Prostate, Lung, Colorectal, and Ovarian Cancer Screening Trial–chest-radiography screening group [12, 16]

^d^NIH-AARP (formally known as the American Association of Retired Persons Study of Diet and Health, US-American National Institute of Health) [15]

^e^Cancer Prevention Study 2 [15]

^f^45-up [13]

^e^National Health Interview Survey [15, 26]

### Eligibility by age and sex: standard criteria versus risk models

For this German adult population we found that the prevalence of smoking varies quite strongly with age and by sex, with more current smokers in the younger age groups, more long-term quitters (ex-smokers) in older age groups, and overall a higher prevalence of ever smoking among men than among women. The different prevalence of long-term smoking habits in younger and older age groups can be explained by an increasing percentage of long-term quitters (ex-smokers) in the older age groups, plus, for women, a well-documented trend of increasing smoking prevalence in more recent birth cohorts. Reflecting these age-related differences in the prevalence of current and past smoking, screening eligibility by the NELSON (55-75-10 × 30/15 × 25 − 10), USPSTF/NLST (55–80-75-30-15) or also the more stringent 55-80-40-10 criteria was found to be higher in younger and lowest amongst the oldest age groups. Our analyses show that, compared to using the USPSTF/NLST, NELSON or other criteria selection based on risk estimates shifts eligibility towards individuals with older age and with higher prevalence of self-reported COPD (when considered by the risk model, e.g. PLCO_M2012_), including ex-smokers with longer average quitting times. The most extreme shift towards higher age (Supplemental Fig. 5) was observed for the LLP_2008_ model, a model that ignores the progressive attenuation of lung cancer risk after smoking cessation.

Analyses of data from US cohorts and general population surveys [15], as well as from the NLST trial [37, 38], indicated similar shifts towards older age using risk-targeted screening eligibility based on risk models, and additionally showed that model-based targeting of screening leads to inclusion of participants with more comorbid conditions compared to participants eligible by the original NLST criteria [37, 38, 15]. Therefore efficiency gains may be more modest in terms of overall and quality-adjusted life-years gained [38]. In line with this latter finding, extensive simulation modeling in context of the Cancer Intervention and Surveillance Modeling Network (CISNET) showed that, while the most efficient screening strategies that maximize the mortality reduction irrespective of over-diagnosis screen through age 80 [35], screening strategies that stop at age 75 versus 80 are expected to produce greater efficiency in increasing life years gained per over-diagnosed case [39, 40].

### Limitations of present analyses

Our analyses are subject to some limitations. Firstly, our data modeling and interpretation mostly account for age, sex and smoking histories, whereas data were missing for some model predictor variables such as family predisposition for cancer, history of pneumonia, dust exposure. Incomplete entries in the available data were filled with simple imputation, aimed at presenting cautionary conservative findings. Secondly, our analyses cover the time period of 2008–2013. Due to continuous changes in the demographic population structure and in the prevalence of current and past smoking among men and women in different age groups, the estimated percentages and total numbers of individuals eligible for screening according to analyses of the 2008–2013 data may not perfectly represent the exact population percentages and numbers at the time (most likely 2021–2022) when CT screening may be introduced in Germany.

### Implications of risk prediction models for screening policy and further research

Recommended criteria for lung cancer screening eligibility so far have been defined mostly in terms of lower and upper age limits, combined with basic summary measures of lifetime cumulative smoking exposure and maximum time since smoking cessation, and have been defined mostly as extensions from criteria used in randomized screening trials, in particular the National Lung Screening Study in the USA (NLST). Judging by the good overall balance between projected reduction in lung cancer mortality and gain in life years (LYG) versus expected biopsies or surgeries for benign lesions and cases of over-diagnosis [35] the US Preventive Services Task Force (USPSTF) defined its recommendation of annual screening for men and women age 55 to 80 (stopping age) with minimally 30 pack years of cumulative lifetime smoking exposure and who have not quitted smoking since more than 15 years (coded: A-55-80-30-15)—a scenario similar to that of the NLST trial (A-55-75-30-15) but with stopping age 80 instead of 75 years [8]. Other US organizations, as well as medical expert organizations in Canada [9], Europe [41] and Germany [42] advocate adhering to the original NLST criteria, i.e. with stopping age 75 instead of 80, in view of limited residual life expectancy and high risks of over-diagnosis at higher age.

Compared to standard criteria based on age limits and summary indices for minimal lifetime smoking exposure, such as USPSTF, NLST or NELSON, studies in North America and Germany have shown that risk prediction models more accurately identify the individuals with the highest lung cancer risks in a given study population. Thus, at equal numbers of individuals to be screened, risk-based strategies identify screening participants with 10–20% greater average risk and hence a smaller number to be screened per lung cancer case. Especially in North America this possible gain in screening efficiency is evoked as key argument for using risk models instead of NLST or USPSTF criteria to determine screening eligibility, while maintaining equivalent target numbers of screening-eligible individuals [12, 15, 16, 25, 26]. With a minimal-risk threshold of about 1.6 or higher—i.e., leading to equivalent numbers of screen-eligible individuals as with NLST or USPSTF criteria (Table 3)—a model-based approach also provides a direct guarantee that each individual screening participants will meet minimal-risk requirements to offset lifelong radiation risks (not directly given by standard eligibility criteria; supplemental Fig. 3), as well a minimal balance of screening benefit over potential harms caused by invasive investigations following false positive screen tests.

The possible downside of using minimal model risk as inclusion criterion is that higher-risk groups thus identified tend to include older subjects compared to NLST, USPSTF or NELSON criteria and, depending on the model used, higher numbers of patients with chronic pulmonary disease, which may limit efficiency gains of screening in terms of life years to be gained and result in more over-diagnosis. Recent quantitative modeling of US data showed that, due to these shifts in risk profiles, risk-based screening strategies requiring similar screens among individuals ages 55–80 years as the USPSTF criteria would avert considerably more lung cancer deaths, but result in only modestly higher life-years gained, while leading to significantly more overdiagnosed cases. Further sensitivity analyses, however, suggested that excluding individuals with limited life expectancies (< 5 years) from screening retains the life-years gained by risk-based screening, while reducing overdiagnosis by about two thirds [43].

Taken together, the results from our and other studies suggest that using a minimal risk estimate, determined by a well-calibrated prediction model, as inclusion criterion may improve the overall efficiency of lung cancer screening, and improve the balance of screening benefits over possible harms caused by radiation or false-positive screen tests, provided that concurrent measures are taken exclude individuals with low (e.g. less than 5-years) residual life expectancy. For Germany, and possibly other European countries, a possible start for introducing CT screening could be to target the ever smokers age 50 to 75 who have a 5-year lung cancer risk above the threshold of about 1.6%, e.g. according to PLCO_M2012_ or another well-calibrated risk model. Based on federal-wide survey data for the years 2008–2013, we find that this target population would encompass about 3.0 million men and women, and that about 40% of all incident lung cancer cases in Germany will occur within this risk set (Supplemental Fig. 4). In view of ongoing and projected changes in the population structure and smoking habits, future screening programs should be implemented with a surveillance system closely monitoring efficiency in strata of age, sex and eligible risk groups, and with continuous monitoring of benefits and harms in terms of reduced mortality and false positive findings. While at first introduction it will be prudent to stop screening at age 75, in view of rapidly growing risks of being over-diagnosed at higher ages, future work may focus on replacing maximum-age limits by using more differentiated models or decision algorithms for predicting individuals’ residual life expectancy, and their potential of gaining a meaningful number of life years in case of early lung cancer detection, e.g. combining basic questionnaire data and clinical fitness indicators.

Finally, we recommend that simulation modeling should be performed specifically for the German population context to estimate expected benefits and harms of screening scenarios with eligibility based on absolute model risk thresholds, as compared to more concise eligibility criteria, and to examine the possible consequences of electing more individuals of older age and with more frequent history of COPD and other comorbidities. Furthermore, as recent studies suggest that CT screening may lead to greater relative mortality reduction among women than among men, due to more frequent occurrence of non-small cell and non-squamous tumors among women [2, 7, 44, 45] simulation studies should also address the question whether screening efficiency can be further optimized using sex-specific eligibility screening criteria or risk thresholds.

## Electronic supplementary material

Below is the link to the electronic supplementary material.

Supplementary material 1 (DOCX 1435 kb)
