# Supplementary material for: Risk prediction models versus simplified selection criteria to determine eligibility for lung cancer screening: an analysis of German federal-wide survey and incidence data
Source: Eur J Epidemiol. 2020 Jun 27;35(10):899–912. doi: 10.1007/s10654-020-00657-w (PMC7524688; doi:10.1007/s10654-020-00657-w)
Supplement: Supplementary file 1 — Supplementary material 1 (DOCX 1435 kb) [file 10654_2020_657_MOESM1_ESM.docx]

**Supplementary material to manuscript** by A. Hüsing, R. Kaaks:

**Risk prediction models versus simplified selection criteria to determine eligibility for lung cancer screening – an analysis of German federal-wide survey and incidence data**

Submitted for publication to European Journal of Epidemiology in 2019

**Table of Content Page**

| **Supplemental Table 1.**  Eligibility criteria employed in lung cancer screening trials, observed lung cancer incidence at baseline and during follow-up screens, % eligible among smoking German adults age 50-80 (GEDA 2008-2013) | **2** |
| --- | --- |
| **Supplemental Table 2a.** Components of selected lung cancer risk models | **3** |
| **Supplemental Table 2 b.**  Detailed risk factor coefficients in selected lung cancer risk models | **4** |
| **Supplemental Table 3.**  Summary of reported performance of risk models | **5** |
| **Supplemental Figure 1.** Distribution of exposure to tobacco smoking in ever-smoking men and women | **6** |
| **Supplemental Figure 2.** Distribution of 5-year-lung cancer risk estimates from 4 investigated models in the general German smoking population in age-groups of men and women between 50 and 80 years, according to the survey sample data from GEDA 2008-2013 (average) | **7** |
| **Supplemental Figure 3.** Distribution of 5-year risk estimates from different models in groups of individuals eligible or not according to criteria in ever-smoking adults age 50-79. | **8** |
| **Supplemental** **Table 4.** Pearson correlation coefficients between different model estimates; GEDA data from 2008-2013, for smokers aged 50-79, by gender | **9** |
| **Supplemental Figure 4.** Predictive capacity of lung cancer risk models projected for the population of ever-smoking German adults age 50-75 | **10** |
| **Supplemental Figure 5.** Eligibility to smoking criteria or risk estimate above threshold identifying equal number of persons among ever-smokers in the German general population (GEDA 2008-2013) | **11** |
| **Supplemental Table 5.** Personal features of ever-smoker age 50-79 selected by eligibility criteria or by risk-threshold identifying same number of subjects to be screened, or both | **14** |
| **Supplemental Figure 6.** Proxy-Calibration of average age-specific expected case-numbers in smoking men and women from 1-year risk estimates for GEDA (2008-09, 2009-10, 2012-13) and as reported from German nation-wide registry data (RKI 2009, 2010, 2013, average), after correcting for proportion of cases attributable to non-smokers | **16** |

**Supplemental Table 1. Eligibility criteria employed in lung cancer screening trials, observed lung cancer incidence at baseline and during follow-up screens, % eligible among smoking German adults age 50-80 (GEDA 2008-2013)**

| Trial | Eligibility criterion:  ≥ age min - < age max  - ≥ pack-years  - <years since quitting | screened participants (follow-up time) **^a)^** | prevalence at baseline  (%)**^b)^** | incidence total (%)**^c)^** | incidence  projected to 5-yr fup (%) **^d)^** | % eligible German smokers age 50-79 |
| --- | --- | --- | --- | --- | --- | --- |
| NLST | 55-75-30-15 | 26722 (6.5y) | 1.0 | 2.4 | 1.1 | 19.1 |
| ITALUNG | 55-70-20-10 | 1406 (3y) | 1.5 | 2.8 | 2.2 | 19.1 |
| DANTE | 60-75-20-x | 1276 (2.8y) | 2.2 | 4.7 | 4.5 | 23.5 |
| DLCST | 50-70-20-10 (min quitting-age 50) | 2052 (4.8y) | 0.8 | 3.4 | 2.6 | 26.0 |
| LUSI | 50-70-[≥15cpd x ≥25yrs or ≥10cpd x ≥30yrs]-10  50-70-[18.75 or 15]-10 | 2029 (5y) | 1.2 | 3.1 | 2.2 | 31.1 |
| NELSON | 50-75-[≥15cpd x ≥25yrs or ≥10cpd x ≥30yrs]-10  50-75-[18.75 or 15]-10 | 7907 (5y) | 0.9 | 1.6 | 0.7 | 34.4 |

DANTE: the Italian DANTE (Detection and Screening of Early Lung Cancer by Novel Imaging Technology and Molecular Essays) trial; DLCST: the Danish Lung Cancer Screening Trial; ITALUNG: the Italian ITALUNG trial; NELSON: the NEtherlands Leuven Screening ONderzoek (NELSON) trial; LUSI: the German Lung Cancer Screening intervention; NLST: the US National Lung Screening trial.

**a)** duration of follow-up time after baseline-screen in years.

**b)** cases per hundred subjects screened, detected at baseline screen

**c)** cases per hundred subjects screened, detected at any screen

**d)** cases per hundred subjects screened, detected at incidence screens only, projected to general 5 years follow-up time.

**Supplemental Table 2a. Components of selected lung cancer risk models (extracted from supplementary material published by Katki et al. (1))**

| **Prediction Model**  **Risk Factor** | **Bach(2)** | **LLP_2008_ (3)** | **PLCO_M2012_ (4)** | **LCRAT(5)** |
| --- | --- | --- | --- | --- |
| Time-span to Endpoint | 10 years LC | 5 years LC | 6 years LC | 5 years LC |
| Age | Spline | Age and sex-specific incidence rate | Continuous | Log-transformed |
| Gender | Categorical |  | — | Categorical |
| Race/ethnicity | — | — | Categorical | Categorical |
| Education | — | — | Ordinal | Ordinal |
| Body mass index | — | — | Continuous | Categorical  and log-transformed |
| Comorbidities: |  |  |  |  |
| COPD | — | — | Categorical | — |
| Pneumonia | — | Categorical | — | — |
| Emphysema | — | — | — | Categorical |
| Cancer | — | Categorical | Categorical | — |
| Family history of lung cancer | — | Categorical | Categorical | Ordinal |
| Occupat. exposure to |  |  |  |  |
| Asbestos | Categorical | Categorical | — | — |
| Smoking |  |  |  |  |
| Smoking status | — | — | Categorical | — |
| Pack-years | — | — | — | Categorical |
| Cigarettes per day | Spline | — | Continuous | Categorical |
| Years smoked | Spline | Categorical | Continuous | Continuous |
| Years quit smoking | Spline | — | Continuous | Log-transformed |

1. Katki HA, Kovalchik SA, Petito LC, et al. Implications of Nine Risk Prediction Models for Selecting Ever-Smokers for Computed Tomography Lung Cancer Screening. Annals of internal medicine. 2018;169(1):10-9. doi:10.7326/M17-2701

2. Bach PB, Kattan MW, Thornquist MD, et al. Variations in lung cancer risk among smokers. Journal of the National Cancer Institute. 2003;95(6):470-8. doi:10.1093/jnci/95.6.470

3. Cassidy A, Myles JP, van Tongeren M, et al. The LLP risk model: an individual risk prediction model for lung cancer. British journal of cancer. 2008;98(2):270-6. doi:10.1038/sj.bjc.6604158

4. Tammemagi MC, Katki HA, Hocking WG, et al. Selection criteria for lung-cancer screening. N Engl J Med. 2013;368(8):728-36. doi:10.1056/NEJMoa1211776

5. Katki HA, Kovalchik SA, Berg CD, Cheung LC, Chaturvedi AK. Development and Validation of Risk Models to Select Ever-Smokers for CT Lung Cancer Screening. JAMA. 2016;315(21):2300-11. doi:10.1001/jama.2016.6255

**Supplemental Table 2b. Detailed risk factor coefficients in selected lung cancer risk models**

| **Risk Factor Prediction Model** | **Bach(2)** | **LLP_2008_ (3)** | **PLCO_M2012_ (4)** | **LCRAT(5)** |
| --- | --- | --- | --- | --- |
| **beta-coefficients (ln(hr) )** |  |  |  |  |
| Age | Spline | intercept by age&sex | 0.078 x (age-62) | 4.387 x ln(age) |
| Gender: female | -0.0583 |  | — | -0.083 |
| Race/ethnicity | — | — | Categorical | Categorical |
| Education | — | — | -0.0813 (edu-cat-4) | -0.0726 x category |
| Body mass index | — | — | -0.027 x (bmi-27) | -0.713 x ln(bmi)  + 0.058 x (bmi>=18.5) |
| COPD | — | — | 0.355 | — |
| Pneumonia | — | 0.602 | — | — |
| Emphysema | — | — | — | 0.56 |
| Cancer | — | 0.675 | 0.459 | — |
| Family history of  lung cancer | — | 0.703 x early  (onset<60ys),  0.168 x late  (onset>=60ys.) | 0.587 | 0.42 |
| Occupational exposures to |  |  |  |  |
| Asbestos | 0.2154 | 0.634 | — | — |
| Smoking status | — | — | 0.260 ( if current) | — |
| Pack-years (pys) | — | — | — | 0.488(if 30-<40pys), 0.565 x (if 40-<50pys),  0.718 x ( if >=50pys) |
| Cigarettes per day (cpd) | 0.0608 | — | -1.82 x (10/cpd-0.4) | 0.307 x (>1pack/day) |
|  | -0.00015  x( cpd--15)**3 +0.00018  x( cpd--20.2)**3-0.00004  x( cpd--40)**3 |  |  |  |
| Years smoked | 0.114 | 0.769 (if 1-20ys), 1.45 (if 21-40ys), 2.5(if 41-60ys),  2.7 if >60ys | 0.0317 x(no. ys -27) | 0.0200 x ys |

2. Bach PB, Kattan MW, Thornquist MD, et al. Variations in lung cancer risk among smokers. Journal of the National Cancer Institute. 2003;95(6):470-8. doi:10.1093/jnci/95.6.470

3. Cassidy A, Myles JP, van Tongeren M, et al. The LLP risk model: an individual risk prediction model for lung cancer. British journal of cancer. 2008;98(2):270-6. doi:10.1038/sj.bjc.6604158

4. Tammemagi MC, Katki HA, Hocking WG, et al. Selection criteria for lung-cancer screening. N Engl J Med. 2013;368(8):728-36. doi:10.1056/NEJMoa1211776

5. Katki HA, Kovalchik SA, Berg CD, Cheung LC, Chaturvedi AK. Development and Validation of Risk Models to Select Ever-Smokers for CT Lung Cancer Screening. JAMA. 2016;315(21):2300-11. doi:10.1001/jama.2016.6255

**Supplemental Table 3. Summary of reported performance of risk models**

| **Study** | **Age-range** | **N (original cohort)** | **ever-smokers** | **Cases**  **(in ever-smokers)** | **Duration of follow-up** | **BACH (10 yrs)** | | | **LLP**  **(5 years)** | | **PLCO_M2012_**  **(6 years)** | | **LCRAT**  **(5 years)** | |
| --- | --- | --- | --- | --- | --- | --- | --- | --- | --- | --- | --- | --- | --- | --- |
|  |  |  |  |  |  | **E/O** | **AUC** | | **E/O** | **AUC** | **E/O** | **AUC** | **E/O** | **AUC** |
| primary publication (internal validation) | | | |  |  |  | | 0.72 |  | 0.7 |  | 0.7 |  |  |
| NLST-CXR (6) | | 53,452 | 53,452 | 1,925 |  |  | | 0.70 |  | 0.67 |  | 0.71 |  | 0.70 |
| NLST-CT (7) | |  |  |  |  |  | | 0.68 |  | 0.65 |  | 0.68 |  |  |
| PLCO-CXR (6, 7) | |  | 37,332 (40600) | 677 (754) | 75% >=10 ys |  | | 0.78 |  | 0.75 |  | 0.80 |  | 0.80 |
| PLCO-no screening | | |  |  |  |  | | 0.77 |  | 0.75 |  | 0.79 |  |  |
|  |  |  |  |  |  |  | |  |  |  |  |  |  |  |
| NIH-AARP (1) | 50-71 | 566,398 | 337,388 | 11,590 | 77%>= 10ys | 1.00 | | 0.76 | 1.18 | 0.73 | 0.92 | 0.77 | 0.97 | 0.77 |
| NIH-AARPxNLSTcriterion | |  |  |  |  | 0.96 | | 0.65 | 0.96 | 0.63 | 0.96 | 0.66 | 0.99 | 0.66 |
| CPS-II (1) | 40-92 | 184,194 | 72,338 | 2,166 | 67%>=10 ys | 0.97 | | 0.75 | 1.72 | 0.73 | 0.99 | 0.75 | 1.12 | 0.79 |
| CPS-II x NLSTcriterion | |  |  |  |  | 1.03 | | 0.62 | 1.27 | 0.62 | 1.15 | 0.65 | 1.17 | 0.64 |
| 45-up (Australia )(8) | 55-74 |  | 52,979 | 621 | 5+ys |  | |  |  |  |  | 0.78 |  |  |
| EPIC-D (9) | 40-69 | 50,000 | 20,700 | 92 |  | 0.88 | | 0.81 | 1.12 | 0.79 | 1.03 | 0.81 |  |  |

6. Tammemagi MC, Church TR, Hocking WG, et al. Evaluation of the lung cancer risks at which to screen ever- and never-smokers: screening rules applied to the PLCO and NLST cohorts. PLoS medicine. 2014;11(12):e1001764. doi:10.1371/journal.pmed.1001764

7. Ten Haaf K, Jeon J, Tammemagi MC, et al. Risk prediction models for selection of lung cancer screening candidates: A retrospective validation study. PLoS medicine. 2017;14(4):e1002277. doi:10.1371/journal.pmed.1002277

8. Weber M, Yap S, Goldsbury D, et al. Identifying high risk individuals for targeted lung cancer screening: Independent validation of the PLCOm2012 risk prediction tool. International journal of cancer. Journal international du cancer. 2017;141(2):242-53. doi:10.1002/ijc.30673

9. Li K, Husing A, Sookthai D, et al. Selecting High-Risk Individuals for Lung Cancer Screening: A Prospective Evaluation of Existing Risk Models and Eligibility Criteria in the German EPIC Cohort. Cancer prevention research. 2015;8(9):777-85. doi:10.1158/1940-6207.capr-14-0424

**Supplemental Figure 1. Distribution of exposure to tobacco smoking in ever-smoking men and women**

a: Life-time smoking duration (years)
Ever smoking men Ever smoking women


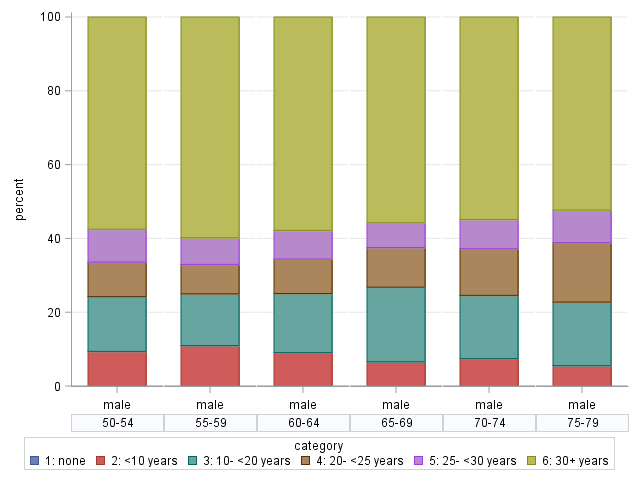

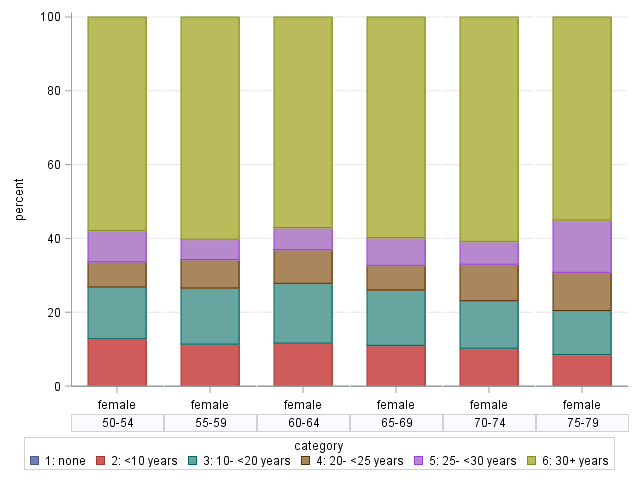


b: Life-time average number of cigarettes per day
Ever smoking men Ever smoking women


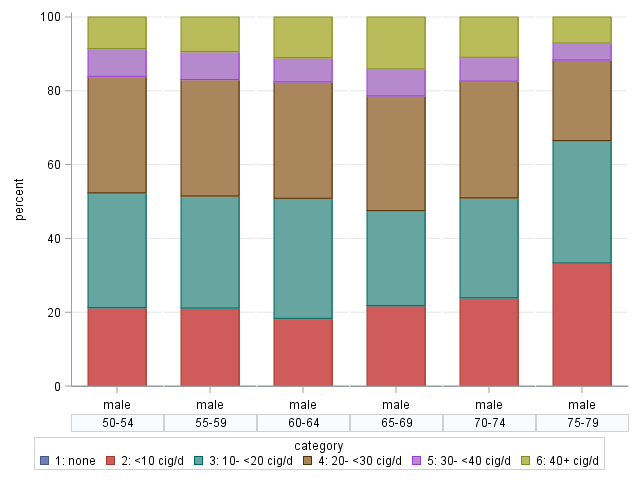

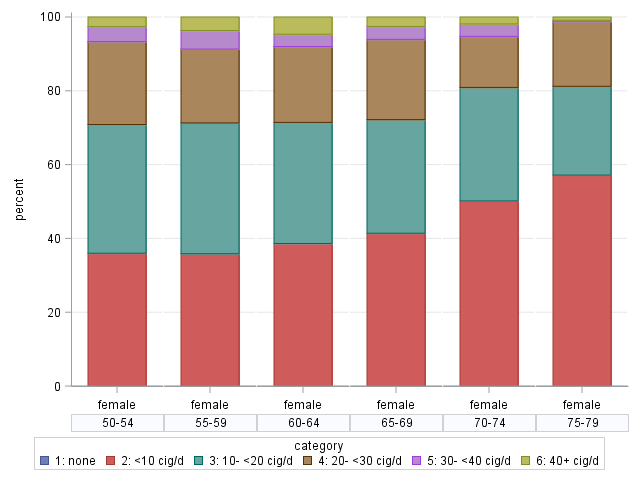


c: Smoking intensity in terms of pack-years
Ever smoking men Ever smoking women


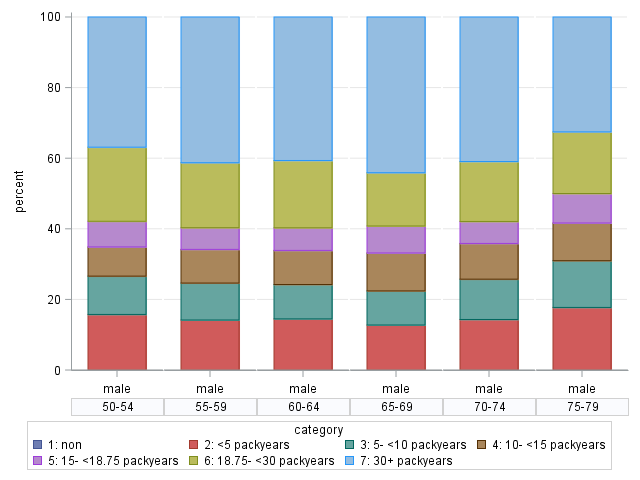

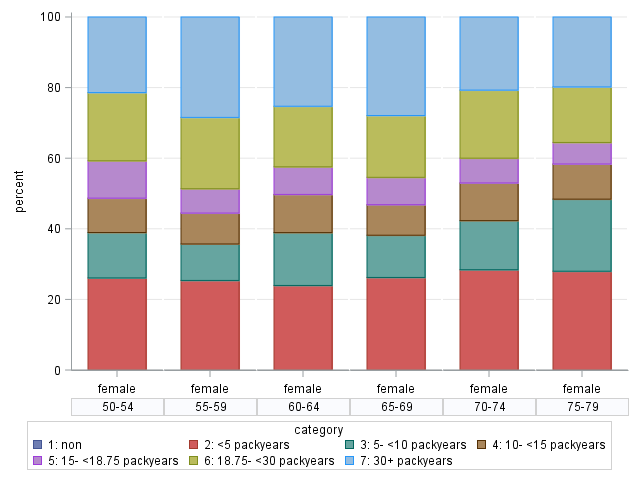


**Supplemental Figure 2. Distribution of 5-year-lung cancer risk estimates from 4 investigated models in the general German smoking population in age-groups of men and women between 50 and 80 years, according to the survey sample data from GEDA 2008-2013 (average)**

| Bach:  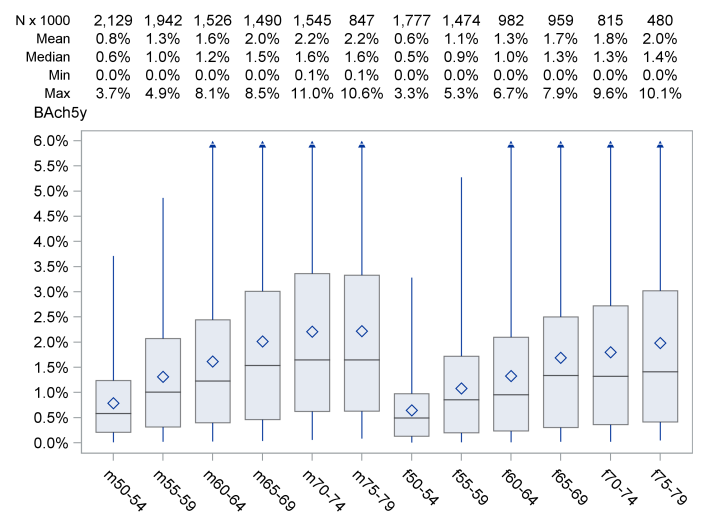 | LCRAT:  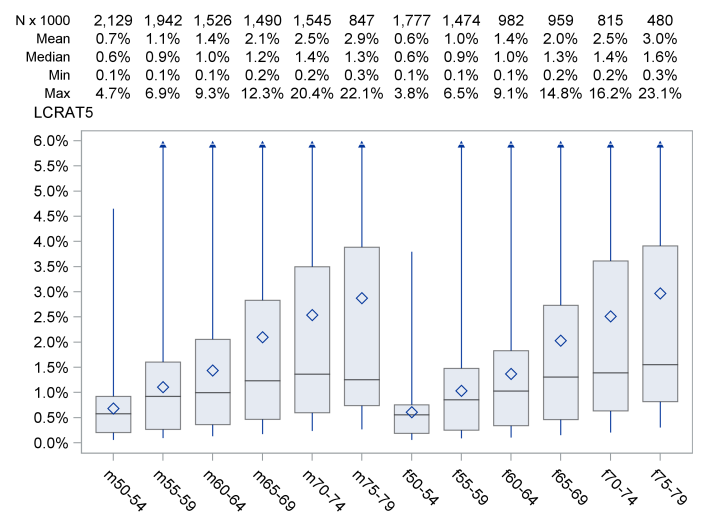 |
| --- | --- |
| LLP:  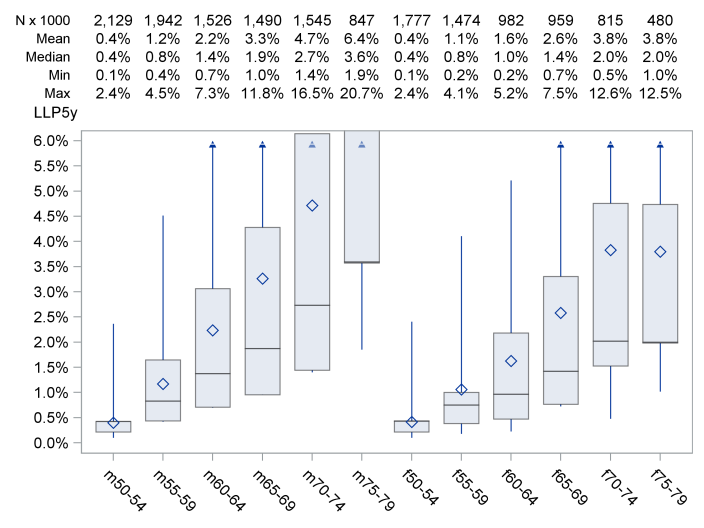 | PLCO_M2012_:  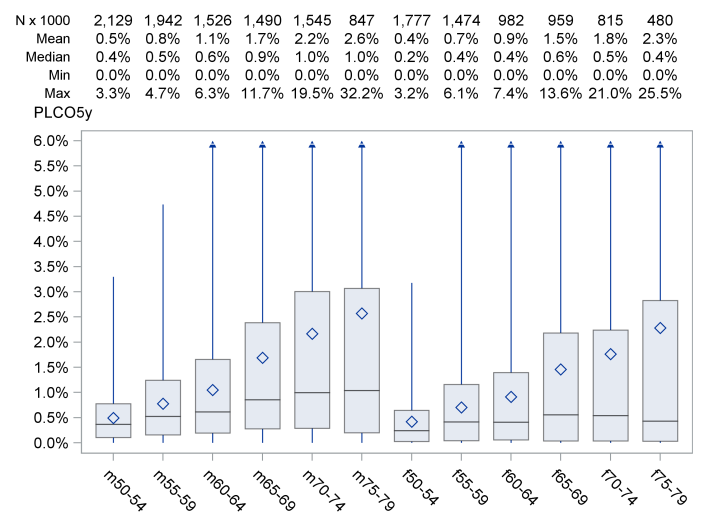 |
|  |  |

**Supplemental Figure 3. Distribution of 5-year risk estimates from different models in groups of individuals eligible or not according to criteria in ever-smoking adults age 50-79.**

| Bach:  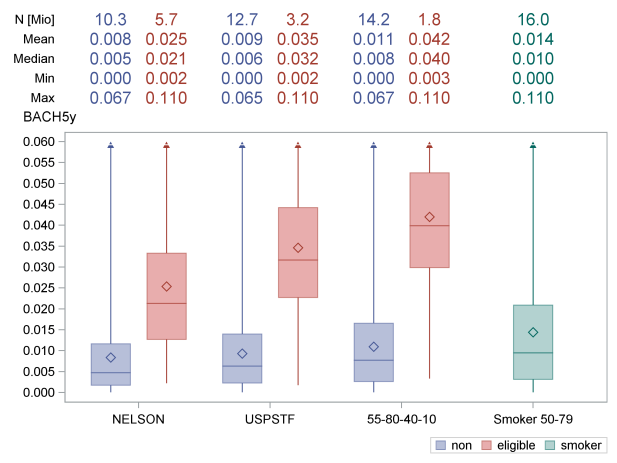 | LLP:  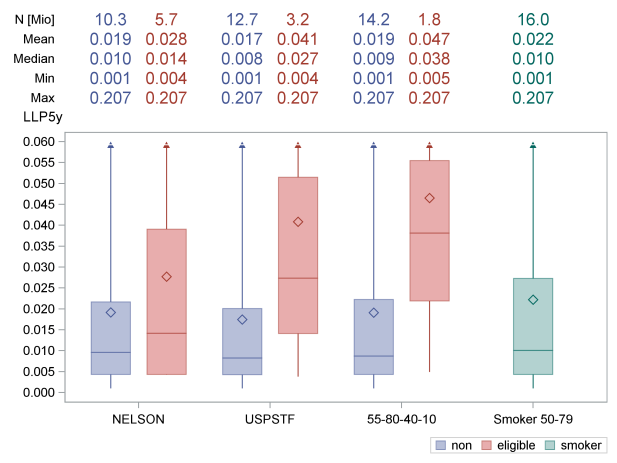 |
| --- | --- |
| PLCO_m2012_:  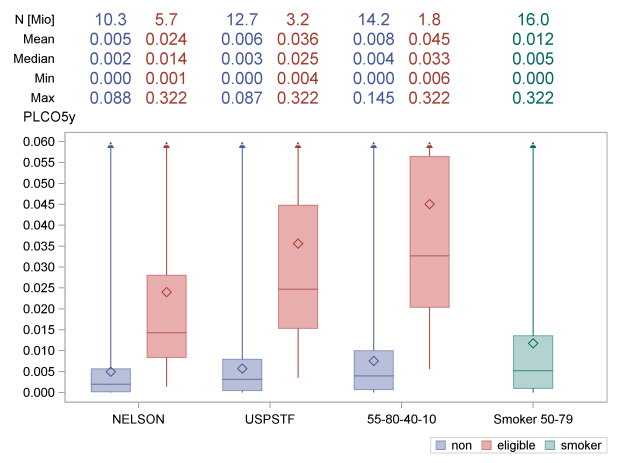 | LCRAT  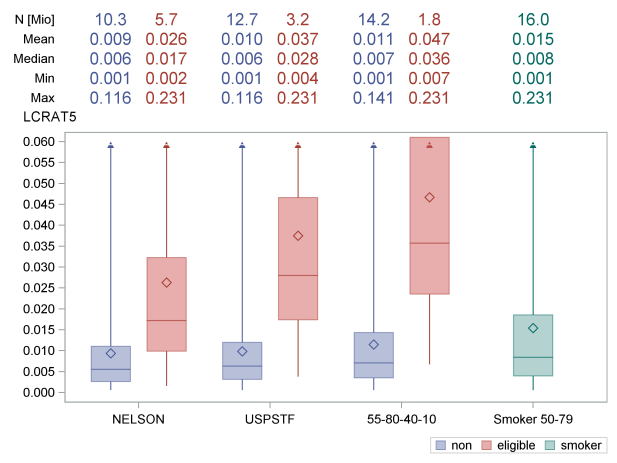 |

**Supplemental** **Table 4. Pearson correlation coefficients between different model estimates; GEDA data from 2008-2013, for smokers aged 50-79, by gender**

|  |  |  |  |  |  |
| --- | --- | --- | --- | --- | --- |
| **men**  **women** | Bach | PLCO_M2012_ | PLCO_ALL2014_ | LCRAT | LLP |
| Bach |  | 84% | 84% | 87% | 67% |
| PLCO_M2012_ | 82% |  | 100% | 89% | 67% |
| PLCO_ALL2014_ | 82% | 100% |  | 88% | 67% |
| LCRAT | 87% | 85% | 85% |  | 70% |
| LLP | 69% | 60% | 61% | 72% |  |

**Supplemental Figure 4. Predictive capacity of lung cancer risk models projected for the population of ever-smoking German adults age 50-75**


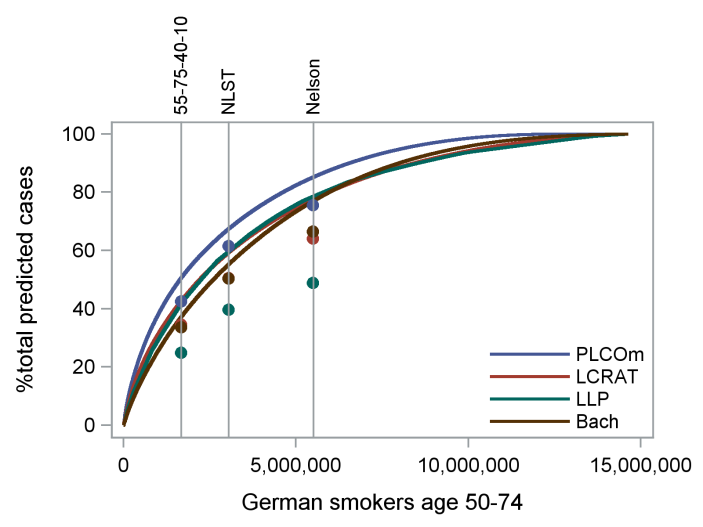

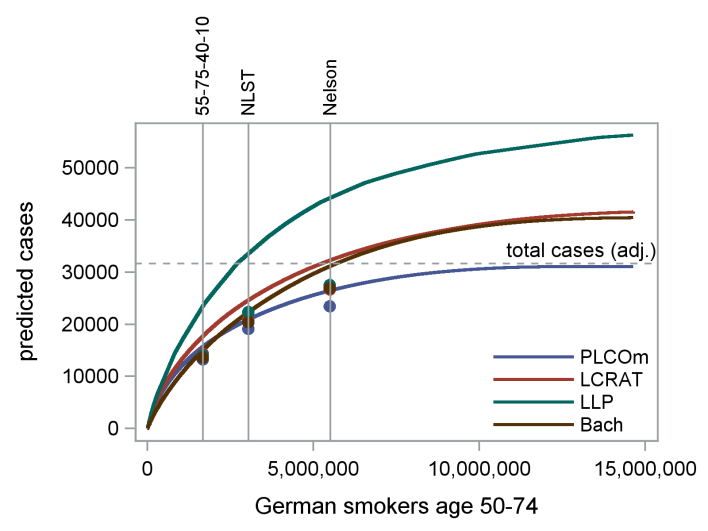


|  |  | **re-calibrated to 31,700 cases** | | | | **according to absolute risk scale** | |
| --- | --- | --- | --- | --- | --- | --- | --- |
|  | % (N) | in eligibles:  % cases  (N, av 5y risk) | model threshold (5y risk) | | above threshold:  % cases  (N, av. 5y risk) | in eligibles:  cases, av 5y risk | above threshold: cases, av 5y risk |
| **NELSON (50-75-10x30/15x25-10)** | | | | | | | |
| Bach | 38% (5.5 Mio) | 66%, ( 21100, 1.9%) | | 1.4% | 77% ( 24400, 2.0%) | 26900, 2.0% | 31200, 2.8% |
| LCRAT | 38% (5.5 Mio) | 64%, ( 20300, 1.8%) | | 1.2% | 78% ( 24600, 2.0%) | 26700, 2.0% | 32300, 2.9% |
| LLP_2008_ | 38% (5.5 Mio) | 49%, ( 15500, 1.4%) | | 1.4% | 79% ( 24900, 2.0%) | 27600, 3.0% | 44300, 4.0% |
| PLCO_M2012_ | 38% (5.5 Mio) | 76%, ( 23900, 2.2%) | | 0.8% | 85% ( 27000, 2.0%) | 23500, 2.0% | 26500, 2.4% |
| **USPSTF (55-75-30-15)** | | | | | | | |
| Bach | 21% (3.0 Mio) | 50%, ( 15900, 2.6%) | | 2.3% | 55% ( 17500, 3.0%) | 20400, 3.0% | 22400, 3.7% |
| LCRAT | 21% (3.0 Mio) | 51%, ( 16000, 2.6%) | | 2.0% | 59% ( 18800, 3.0%) | 21000, 3.0% | 24700, 4.1% |
| LLP_2008_ | 21% (3.0 Mio) | 40%, ( 12600, 2.1%) | | 2.7% | 60% ( 18900, 3.0%) | 22400, 4.0% | 33600, 5.5% |
| PLCO_M2012_ | 21% (3.0 Mio) | 62%, ( 19500, 3.2%) | | 1.5% | 67% ( 21400, 4.0%) | 19200, 3.0% | 21000, 3.4% |
| **55-75-40-10** | | | | | | | |
| Bach | 11% (1.7 Mio) | 34%, (10700, 3.2%) | | 3.2% | 37% (11800, 4.0%) | 13600, 4.0% | 15100, 4.5% |
| LCRAT | 11% (1.7 Mio) | 35%, (11000, 3.3%) | | 3.1% | 43% (13500, 4.0%) | 14400, 4.0% | 17700, 5.3% |
| LLP_2008_ | 11% (1.7 Mio) | 25%, (7900, 2.4%) | | 5.2% | 42% (13200, 4.0%) | 14100, 4.0% | 23500, 7.0% |
| PLCO_M2012_ | 11% (1.7 Mio) | 43%, (13500, 4.0%) | | 2.5% | 51% (16000, 5.0%) | 13200, 4.0% | 15700, 4.7% |

**Supplemental Figure 5: Eligibility to smoking criteria or risk estimate above threshold identifying equal number of persons among ever-smokers in the German general population (GEDA 2008-2013)
a) Eligibility by NLST smoking criterion (50-80-30-15), or by Bach lung-cancer risk.**Men Women

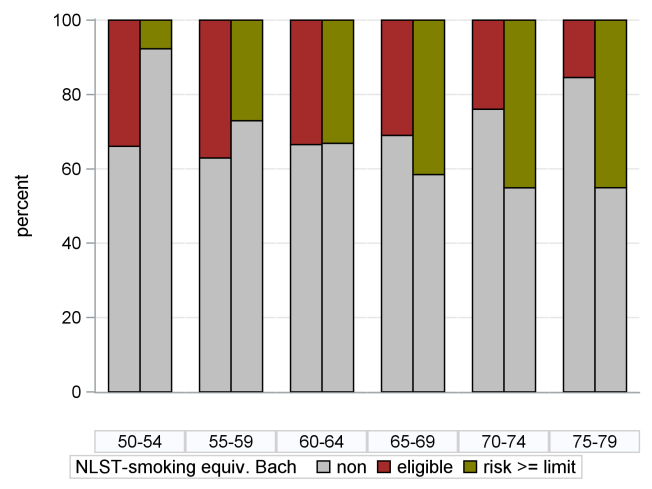

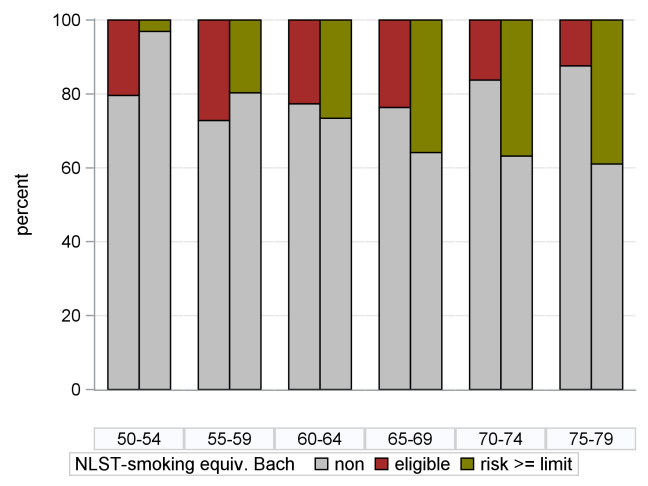


**b) Eligibility by NLST smoking criterion (50-80-30-15), or by LCRAT lung-cancer risk.**Men Women

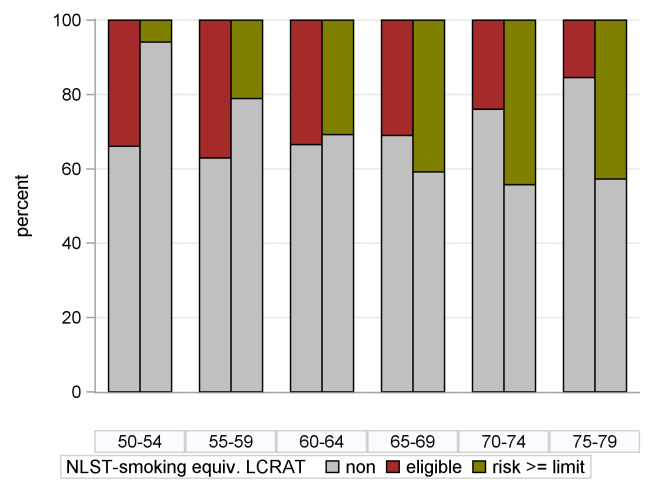

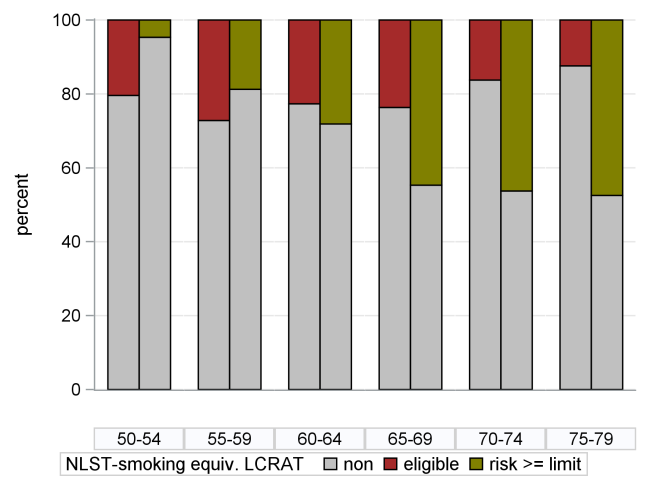


**c) Eligibility by NLST smoking criterion (50-80-30-15), or by LLP lung-cancer risk.**Men Women

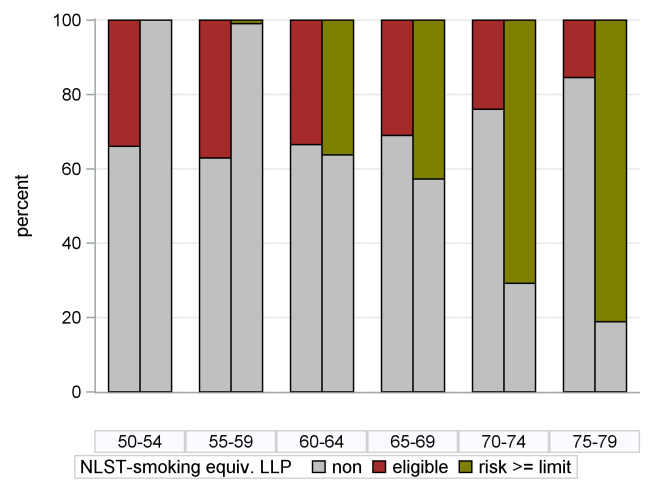

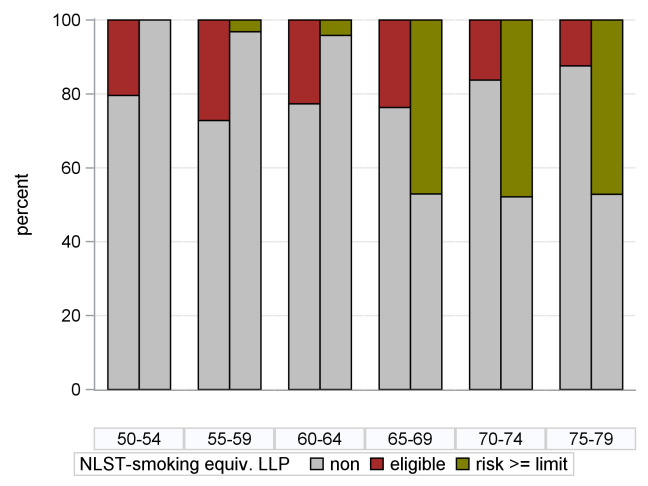


**Supplemental Figure 5 (continued)
d) Eligibility by NELSON smoking criterion (≥10cpd x 30yrs or ≥15cpd x 25yrs, ≤10years quit), or by Bach lung-cancer risk.**Men Women

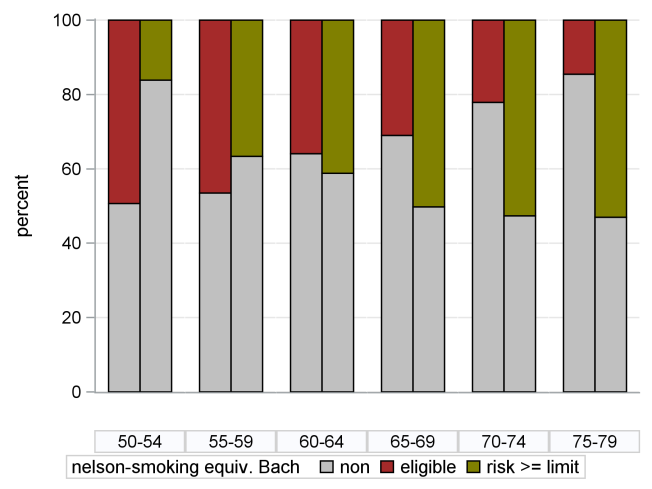

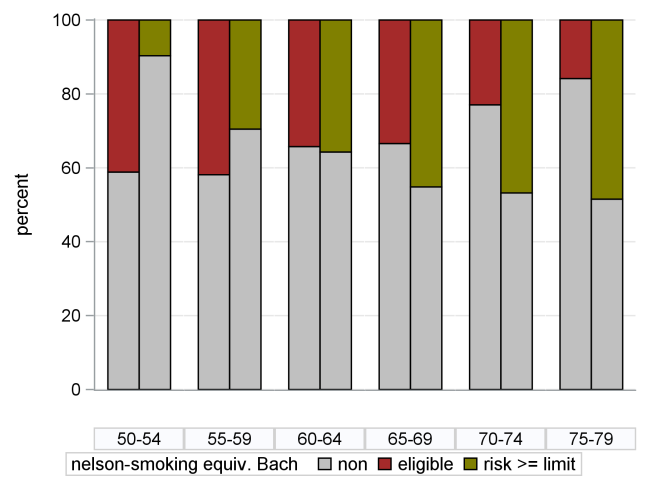


**e) Eligibility by (≥10cpd x 30yrs or ≥15cpd x 25yrs, ≤10years quit), or by LCRAT lung-cancer risk.**Men Women

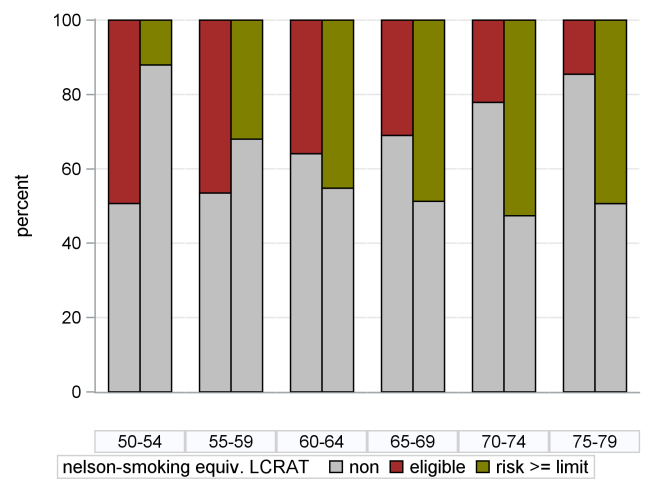

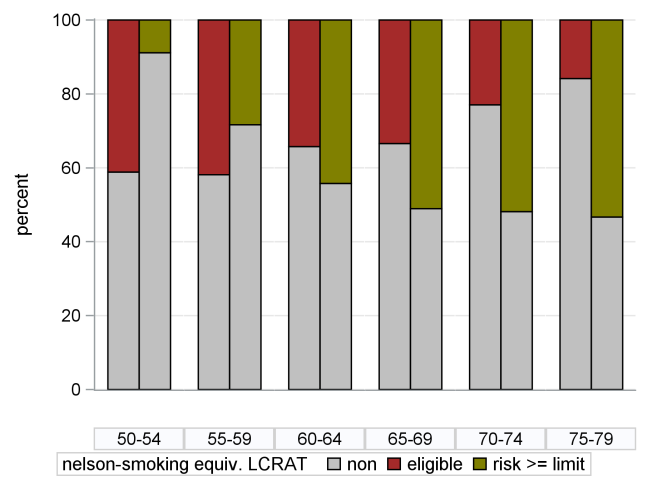


**f) Eligibility by (≥10cpd x 30yrs or ≥15cpd x 25yrs, ≤10years quit), or by LLP lung-cancer risk.**Men Women

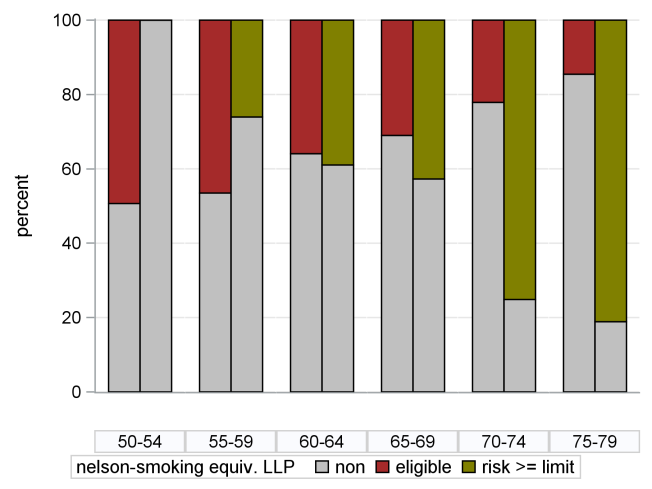

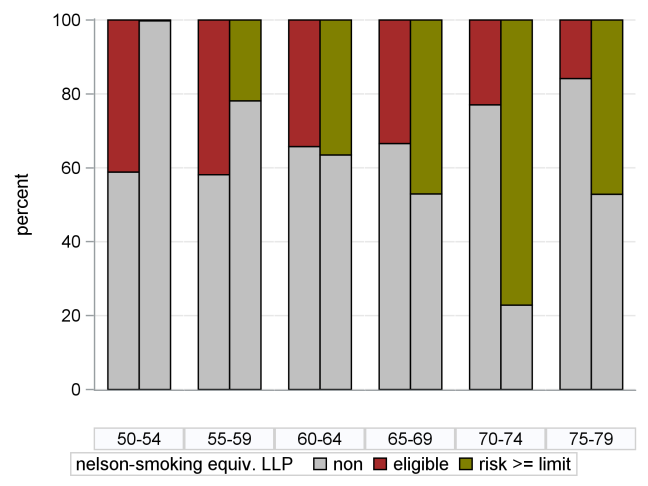


**Supplemental Figure 5. (continued)
g) Eligibility criterion 50-80-40-10, or by Bach lung-cancer risk.**Men Women

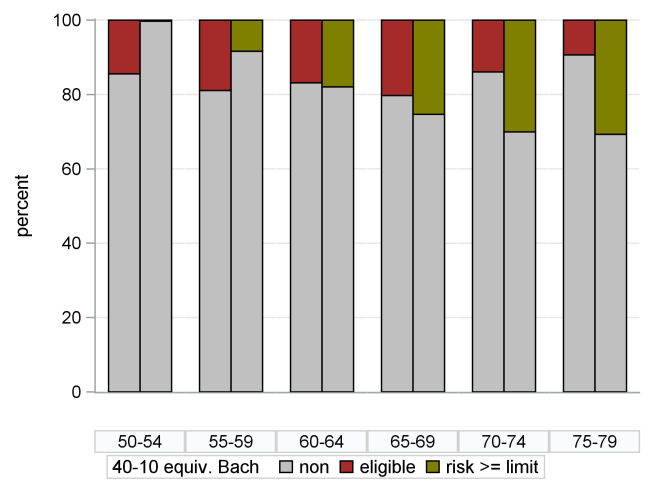

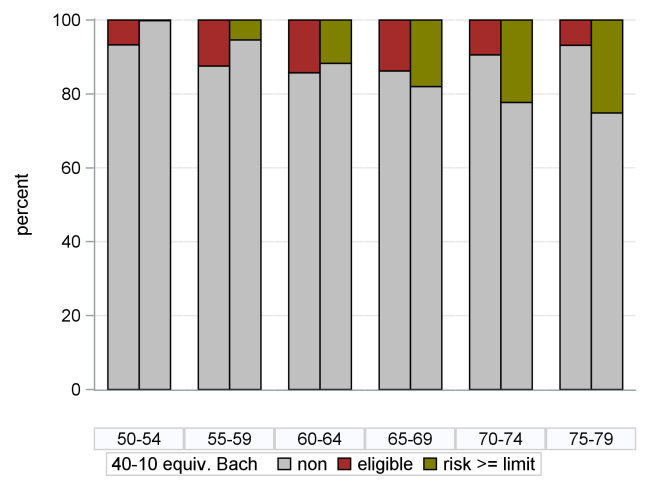


**h) Eligibility by criterion 50-80-40-10, or by LCRAT lung-cancer risk.**Men Women

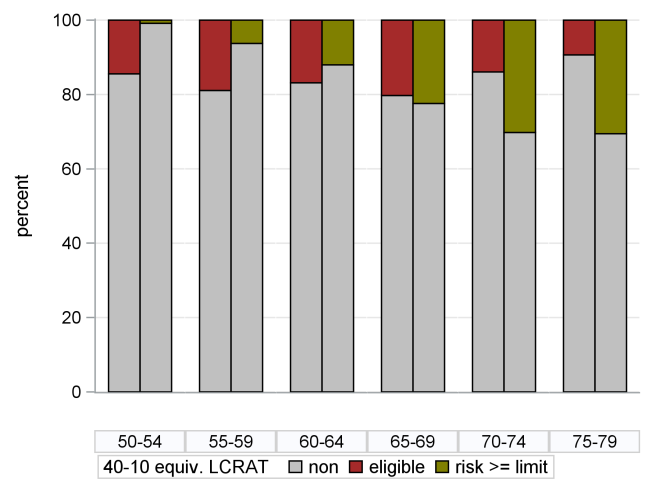

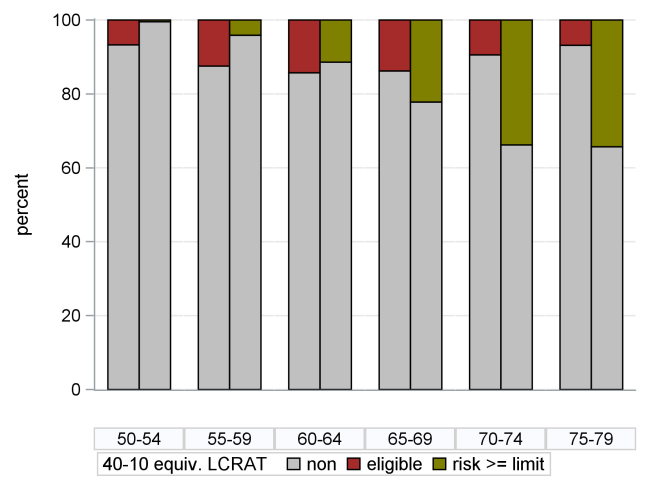


**i) Eligibility by criterion 50-80-40-10, or by LLP lung-cancer risk.**Men Women

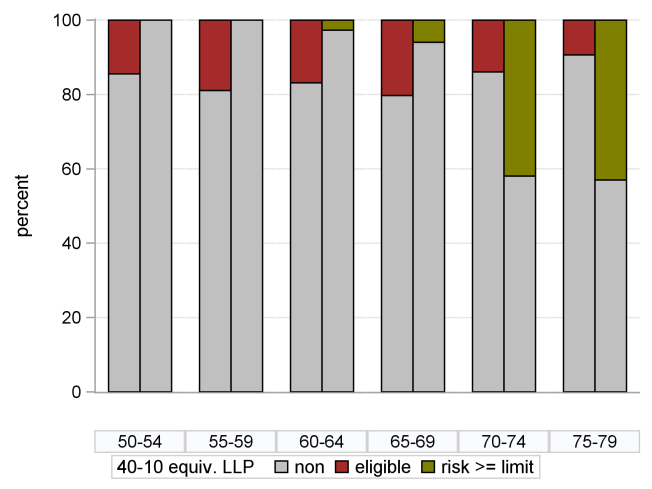

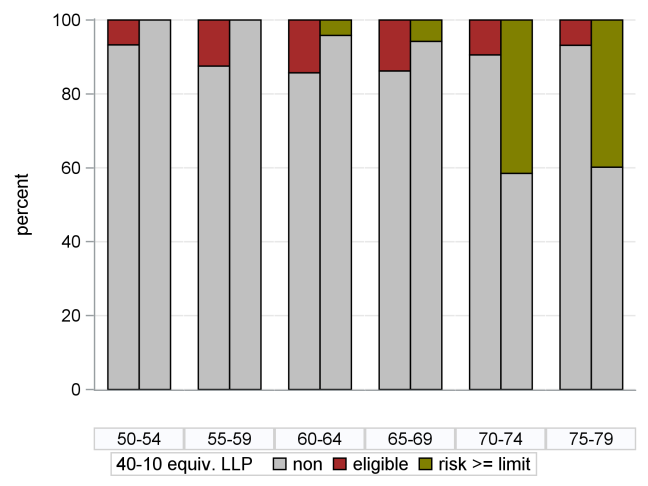


**Supplemental Table 5 Personal features of ever-smoker age 50-79 selected by eligibility criteria or by risk-threshold identifying same number of subjects to be screened, or both**

|  | **USPSTF(55-80-30-15)+,**  **Bach-risk < 2.4%** | **USPSTF(55-80-30-15)--,**  **Bach-risk ≥ 2.4%** | **USPSTF(55-80-30-15)+,**  **Bach-risk ≥ 2.4%** |
| --- | --- | --- | --- |
| male | 64% ( 0.6) | 62% ( 0.6) | 69% ( 1.6) |
| female | 36% ( 0.3) | 38% ( 0.4) | 31% ( 0.7) |
| current smoker | 30% ( 0.3) | 64% ( 0.6) | 71% ( 1.6) |
| former smoker | 70% ( 0.7) | 36% ( 0.3) | 29% ( 0.7) |
| COPD/emphysema | 14% ( 0.1) | 15% ( 0.1) | 17% ( 0.4) |
| age (ys) | 60.1 (59.7 - 60.5) | 70.7 (70.0 - 71.4) | 66.0 (65.5 - 66.4) |
| below age 55 | 0 | 10% ( 0.1) | 0 |
| smoking history (ys) | 36.3 (35.9 - 36.7) | 46.8 (45.9 - 47.7) | 46.8 (46.5 - 47.2) |
| lifetime average cigarettes per day | 23.6 (22.5 - 24.7) | 17.4 (15.9 - 18.9) | 23.7 (23.0 - 24.3) |
| lifetime cigarette consumption (packyears) | 41.8 (40.0 - 43.7) | 35.7 (33.2 - 38.2) | 54.6 (53.1 - 56.0) |
| years since quitting | 5.6 ( 5.1 - 6.1) | 6.9 ( 6.0 - 7.9) | 2.1 ( 1.8 - 2.3) |
| body-mass-index | 27.9 (27.5 - 28.3) | 26.9 (26.4 - 27.4) | 26.9 (26.6 - 27.2) |
| education (US coding) | 3.0 ( 2.9 - 3.2) | 2.7 ( 2.6 - 2.8) | 2.8 ( 2.8 - 2.9) |
|  | **USPSTF(55-80-30-15)+,**  **LCRAT-risk < 2.2%** | **USPSTF(55-80-30-15)--,**  **LCRAT-risk ≥ 2.2%** | **USPSTF(55-80-30-15)+,**  **LCRAT-risk ≥ 2.2%** |
| male | 67% ( 0.8) | 53% ( 0.6) | 68% ( 1.4) |
| female | 33% ( 0.4) | 47% ( 0.6) | 32% ( 0.6) |
| current smoker | 38% ( 0.5) | 79% ( 1.0) | 72% ( 1.5) |
| former smoker | 62% ( 0.8) | 21% ( 0.3) | 28% ( 0.6) |
| COPD/emphysema | 4% ( 0.1) | 27% ( 0.3) | 23% ( 0.5) |
| age (ys) | 60.3 (59.9 - 60.6) | 70.7 (70.1 - 71.3) | 66.7 (66.2 - 67.1) |
| below age 55 | 0 | 6% ( 0.1) | 0 |
| smoking history (ys) | 37.9 (37.5 - 38.3) | 47.5 (46.8 - 48.3) | 47.3 (46.9 - 47.8) |
| lifetime average cigarettes per day | 23.7 (22.8 - 24.7) | 10.7 ( 9.6 - 11.8) | 23.6 (22.9 - 24.3) |
| lifetime cigarette consumption (packyears) | 44.1 (42.4 - 45.8) | 22.5 (20.7 - 24.4) | 55.0 (53.4 - 56.5) |
| years since quitting | 5.1 ( 4.7 - 5.6) | 3.2 ( 2.6 - 3.9) | 1.9 ( 1.6 - 2.1) |
| body-mass-index | 28.4 (28.1 - 28.8) | 25.4 (25.1 - 25.8) | 26.5 (26.1 - 26.8) |
| education (US coding) | 3.2 ( 3.1 - 3.3) | 2.5 ( 2.4 - 2.6) | 2.7 ( 2.7 - 2.8) |

**Supplemental Table 5 (continued)**

| **% (N[Mio]) or**  **mean (95%CI)** | **NELSON+,**  **PLCO_M2012_ - risk < 0.96%** | **NELSON -,**  **PLCO_M2012_ - risk ≥ 0.96%** | **NELSON +,**  **PLCO_M2012_ - risk ≥ 0.96%** | |
| --- | --- | --- | --- | --- |
|  | 100% (1.8Mio) | 100% (1.8Mio) | 100% (3.7Mio) | |
| male | 58% (1.0Mio) | 70% (1.2Mio) | 61% (2.3Mio) | |
| female | 42% (0.7Mio) | 30% (0.5Mio) | 39% (1.5Mio) | |
| current smoker | 62% (1.1Mio) | 19% (0.3Mio) | 75% (2.8Mio) | |
| former smoker | 38% (0.7Mio) | 81% (1.4Mio) | 25% (0.9Mio) | |
| COPD/emphysema | 6% (0.1Mio) | 19% (0.3Mio) | 19% (0.7Mio) | |
| age (ys) | 54.5 (54.3 - 54.7) | 72.3 (72.0 - 72.7) | 61.9 (61.6 - 62.3) | |
| below age 55 | 0 |  | 0 |  |
| smoking history (ys) | 35.2 (34.9 - 35.5) | 37.1 (36.2 - 38.1) | 43.2 (42.9 - 43.6) | |
| lifetime average cigarettes per day | 16.2 (15.7 - 16.7) | 25.0 (23.8 - 26.2) | 21.7 (21.1 - 22.2) | |
| lifetime cigarette consumption (packyears) | 27.8 (27.1 - 28.5) | 42.0 (40.2 - 43.8) | 46.2 (45.1 - 47.4) | |
| years since quitting | 2.1 (1.9 - 2.3) | 16.4 (15.5 - 17.3) | 1.2 (1.1 - 1.3) | |
| body-mass-index | 27.5 (27.2 - 27.8) | 27.5 (27.1 - 27.8) | 26.3 (26.1 - 26.5) | |
| education (US coding) | 3.2 (3.1 - 3.2) | 2.8 (2.7 - 2.9) | 2.8 (2.7 - 2.8) | |
|  | **55-80-40-10+,**  **PLCO_M2012_ - risk < 2.7%** | **55-80-40-10 --,**  **PLCO_M2012_ - risk ≥ 2.7%** | **55-80-40-10 +,**  **PLCO_M2012_ - risk ≥ 2.7%** | |
| male | 69% (0.5Mio) | 61% (0.4Mio) | 68% (0.7Mio) | |
| female | 31% (0.2Mio) | 39% (0.3Mio) | 32% (0.3Mio) | |
| current smoker | 55% (0.4Mio) | 50% (0.4Mio) | 72% (0.8Mio) | |
| former smoker | 45% (0.3Mio) | 50% (0.4Mio) | 28% (0.3Mio) | |
| COPD/emphysema | 9% (0.1Mio) | 27% (0.2Mio) | 22% (0.Mio) | |
| age (ys) | 59.7 (59.4 - 60.0) | 72.5 (71.9 - 73.1) | 68.3 (67.8 - 68.8) | |
| below age 55 | 0 |  | 0 | |
| smoking history (ys) | 41.3 (40.9 - 41.6) | 46.3 (45.4 - 47.1) | 49.8 (49.2 - 50.3) | |
| lifetime average cigarettes per day | 27.2 (26.1 - 28.3) | 19.5 (18.1 - 20.8) | 25.5 (24.6 - 26.5) | |
| lifetime cigarette consumption (packyears) | 55.1 (53.0 - 57.3) | 42.5 (40.0 - 45.1) | 62.5 (60.3 - 64.6) | |
| years since quitting | 2.2 (1.9 - 2.5) | 7.1 (6.1 - 8.1) | 1.4 (1.1 - 1.6) | |
| body-mass-index | 28.6 (28.0 - 29.1) | 25.8 (25.3 - 26.4) | 26.3 (25.8 - 26.7) | |
| education (US coding) | 3.1 (3.0 - 3.3) | 2.5 (2.4 - 2.7) | 2.7 (2.5 - 2.8) | |

**Supplemental Figure 6. Proxy-Calibration of average age-specific expected case-numbers of lung cancer in smoking men and women from 1-year risk estimates for GEDA (2008-09, 2009-10, 2012-13) and as reported from German nation-wide registry data (RKI 2009, 2010, 2013, average), after correcting for proportion of cases attributable to non-smokers***

| Bach:  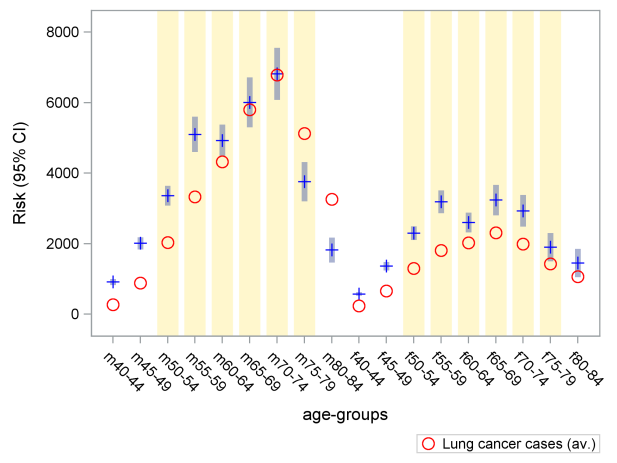 | LLP:  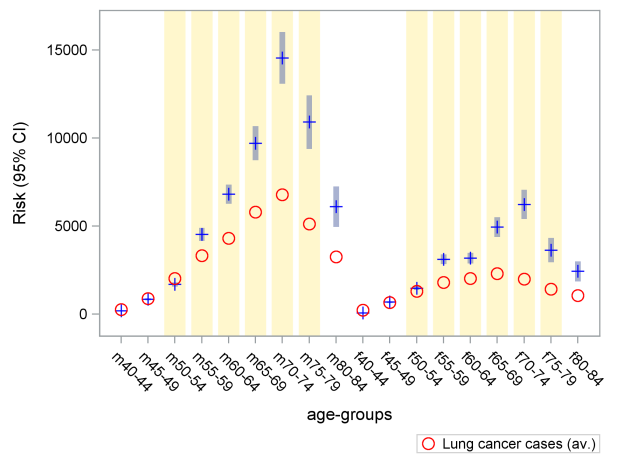 |
| --- | --- |
| PLCO_m2012_:  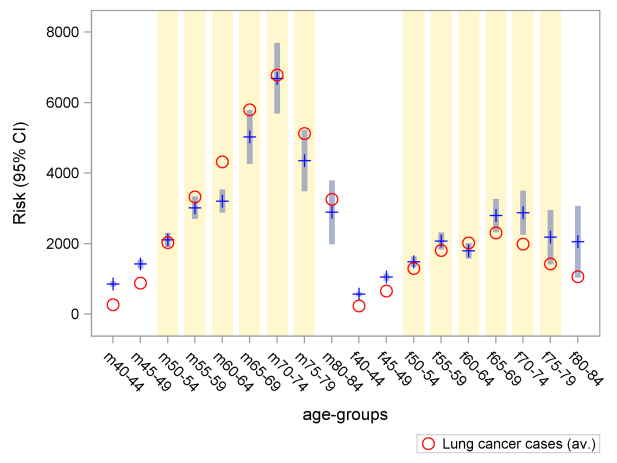 | LCRAT  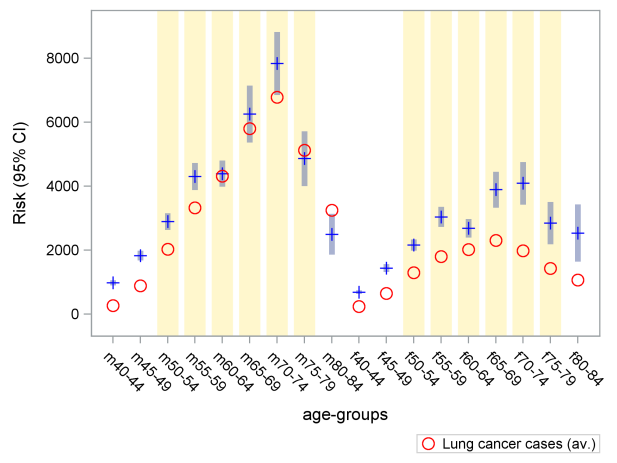 |
| Not only smokers, but total population estimates from PLCO-model including non-smokers, compared with full incidence | |
| PLCO_ALL2014_:  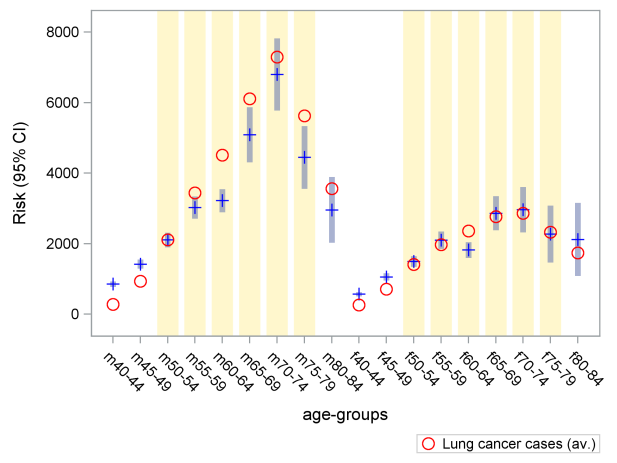 |  |

*PLCO_ALL2014_ model was used to project cases among smokers and nonsmokers according to GEDA (sum of risk estimates), and the originally reported number of cases was reduced by the time, sex- and age-specific proportion of cases projected among non-smokers.
